# National, regional, and global causes of mortality in 5–19-year-olds from 2000 to 2019: a systematic analysis

**DOI:** 10.1016/S2214-109X(21)00566-0

**Published:** 2022-02-15

**Authors:** Li Liu, Francisco Villavicencio, Diana Yeung, Jamie Perin, Gerard Lopez, Kathleen L Strong, Robert E Black

**Affiliations:** a Department of Population, Family, and Reproductive Health, Johns Hopkins Bloomberg School of Public Health, Baltimore, MD, USA; b Department of International Health, Johns Hopkins Bloomberg School of Public Health, Baltimore, MD, USA; c Centre for Demographic Studies, Universitat Autònoma de Barcelona, Barcelona, Spain; d Department of Maternal, Newborn, Child and Adolescent Health and Ageing, WHO, Geneva, Switzerland

## Abstract

**Background:**

Investments in the survival of older children and adolescents (aged 5–19 years) bring triple dividends for now, their future, and the next generation. However, 1·5 million deaths occurred in this age group globally in 2019, nearly all from preventable causes. To better focus the attention of the global community on improving survival of children and adolescents and to guide effective policy and programmes, sound and timely cause of death data are crucial, but often scarce.

**Methods:**

In this systematic analysis, we provide updated time-series for 2000–19 of national, regional, and global cause of death estimates for 5–19-year-olds with age-sex disaggregation. We estimated separately for countries with high versus low mortality, by data availability, and for four age-sex groups (5–9-year-olds [both sexes], 10–14-year-olds [both sexes], 15–19-year-old females, and 15–19-year-old males). Only studies reporting at least two causes of death were included in our analysis. We obtained empirical cause of death data through systematic review, known investigator tracing, and acquisition of known national and subnational cause of death studies. We adapted the Bayesian Least Absolute Shrinkage and Selection Operator approach to address data scarcity, enhance covariate selection, produce more robust estimates, offer increased flexibility, allow country random effects, propagate coherent uncertainty, and improve model stability. We harmonised all-cause mortality estimates with the UN Inter-agency Group for Child Mortality Estimation and systematically integrated single cause estimates as needed from WHO and UNAIDS.

**Findings:**

In 2019, the global leading specific causes of death were road traffic injuries (115 843 [95% uncertainty interval 110 672–125 054] deaths; 7·8% [7·5–8·1]); neoplasms (95 401 [90 744–104 812]; 6·4% [6·1–6·8]); malaria (81 516 [72 150–94 477]; 5·5% [4·9–6·2]); drowning (77 460 [72 474–85 952]; 5·2% [4·9–5·5]); and diarrhoea (72 679 [66 599–82 002], 4·9% [4·5–5·3]). The leading causes varied substantially across regions. The contribution of communicable, maternal, perinatal, and nutritional conditions declined with age, whereas the number of deaths associated with injuries increased. The leading causes of death were diarrhoea (51 630 [47 206–56 235] deaths; 10·0% [9·5–10·5]) in 5–9-year-olds; malaria (31 587 [23 940–43 116]; 8·6% [6·6–10·4]) in 10–14-year-olds; self-harm (32 646 [29 530–36 416]; 13·4% [12·6–14·3]) in 15–19-year-old females; and road traffic injuries (48 757 [45 692–52 625]; 13·9% [13·3–14·3]) in 15–19-year-old males. Widespread declines in cause-specific mortality were estimated across age-sex groups and geographies in 2000–19, with few exceptions like collective violence.

**Interpretation:**

Child and adolescent survival needs focused attention. To translate the vision into actions, more investments in the health information infrastructure for cause of death and in the related life-saving interventions are needed.

**Funding:**

Bill & Melinda Gates Foundation and WHO.

## Introduction

Older children and adolescents (aged 5–19 years) make up a quarter of the world population.^1^ Investments in the survival and health of this age group bring triple dividends: their health now, their future health, and the health of the next generation.^2^, ^3^ However, 1·48 million deaths occurred in this age group globally in 2019,^4^ nearly all from preventable causes.^5^ Following the achievements made in reducing mortality in children younger than 5 years in the Millennium Development Goals era (2000–15), the survival of older children and adolescents has gained attention. Sustainable Development Goal 3 emphasises healthy lives and wellbeing for all ages, and has targets for reducing premature mortality from maternal, communicable, and non-communicable causes, and injuries for 2016–30.^6^, ^7^ The Global Strategy for Women's, Children's, and Adolescent's Health (2016–30) has survival as a main objective and adolescent mortality as one of its 16 core indicators.^8^, ^9^ These and other global initiatives^10^, ^11^, ^12^ have recognised that interventions aimed at these crucial years can lead to far-reaching, multisectoral benefits in promoting lifetime survival and health, alleviating poverty, and stimulating economies.^13^

To better focus the attention of the global community on improving survival of older children and adolescents and to guide effective policy and programmes, sound and timely cause of death data are needed. However, these data are scarce in low-income and middle-income countries (LMICs) due to incomplete or non-existing civil registration and vital statistics systems.^14^ Compared with children younger than 5 years, national data on cause of death in older children and adolescents are particularly scarce due to poor attention, commitment, and resources. We fill this important gap by providing cause of death estimates for 5–19-year-olds at national, regional, and global levels through a systematic analysis.

**Research in context**

**Evidence before this study**

To fill the crucial knowledge gap regarding causes of deaths in older children and adolescents (aged 5–19 years), several estimation exercises have been done. We searched PubMed, Scopus, EMBASE, Web of Science, Global Health Index Medicus, Global Health OVID, IndMed, PAHO, Popline, and Cochrane for studies published between Jan 1, 1980 and Dec 31, 2017, using the search terms (appendix pp 11–12) related to causes of deaths, ages 5–19, and high mortality countries. Nationally representative data have been used to examine causes of deaths in 5–14-year-olds in China, India, Brazil, and Mexico annually for 2005–16. However, this only covered about 40% of the global population aged 5–14 years. The Global Burden of Disease study produced estimates for all countries with time-series. However, it used multiple sources of cause of death data non-differentially, including those from vital registration and verbal autopsy studies, and those from general and special populations. A third study is the Global Health Estimates routinely updated by WHO. Using independent life tables, Global Health Estimates combined WHO single cause programme estimates where available and Global Burden of Disease estimates to generate cause of death estimates for all ages, including older children and adolescents. The limitations associated with the Global Burden of Disease estimates are also the case for Global Health Estimates.

**Added value of this study**

We provide independent cause of death estimates for 5–19-year-olds as a whole, 5–9-year olds and 10–14-year olds of both sexes, 15–19-year-old females, and 15–19-year-old males at national, regional, and global levels. We estimate separately for countries with low and high mortality burden. For the low mortality countries, we use high-quality vital registration data as is or use them as model inputs. For the high mortality countries, we use verbal autopsy studies reporting at least two causes of death from the general population as model inputs.

**Implications of all the available evidence**

Older children and adolescent causes of deaths are under-studied. Severe and persistent empirical data gaps exist. More research is needed to fill these gaps. Based on current research, focused attention on survival in 5–19-year-olds is particularly needed during the Sustainable Development Goal era, in which many global development priorities are competing.

## Methods

### Overview

We provide annual cause of death estimates for 5–19-years-olds from 2000 to 2019 for 195 countries (appendix pp 3–6). We classify the countries into three categories, depending on their data availability and mortality: countries with high-quality vital registration data, countries without high-quality vital registration data and with low mortality, and countries without high-quality vital registration data and with high mortality (appendix pp 3–6). For the last two categories, we estimated cause-specific mortality fractions (CSMFs) using Bayesian multinomial-logistic models. Considering cause of death profile differentials and data availability, we estimated separately for 5–9-year-olds of both sexes, 10–14-year-olds of both sexes, 15–19-year-old females, and 15–19-year-old males. Our cause of death categories (appendix p 7) include any cause of death reported for 3% or more of global deaths in each of the four age-sex groups according to previous estimates.^15^, ^16^ To derive regional mortality estimates, we grouped countries in nine regions according to the UN Inter-agency Group for Child Mortality Estimation (IGME; appendix pp 3–6).^4^, ^17^

### Country modelling

For the 67 high-quality vital registration countries, we used vital registration data as reported or with minor adjustments. Following the same criteria as the WHO's Global Health Estimates (GHE),^16^ high-quality vital registration countries are those with at least 5 years of high quality and complete cause of death data for people aged 15 years and older. For these countries, vital registration data were taken from the WHO mortality database and adjusted for missing years, garbage codes, ill-defined codes, miscoded causes, and programme data.

For the 51 low mortality modelled (LMM) countries, empirical high-quality vital registration data were used as model inputs. To parameterise the model, covariates were selected on the basis of a literature review of sociodemographic variables and risk factors related to cause of deaths for 5–19-year-olds (appendix pp 8–10). We estimated the CSMFs by implementing a Bayesian multinomial-logistic model with country random effects.^18^ Multinomial-logistic models have been previously used to estimate the CSMFs in a frequentist framework.^19^, ^20^ The Bayesian paradigm incorporates three major improvements: a data driven covariate selection process, the inclusion of country random effects when nationally representative data are available, and more coherent uncertainty propagation. Additionally, a Least Absolute Shrinkage and Selection Operator (LASSO)^21^ was used to impose a penalty on the model coefficients, enhance model stability, and prevent overfitting when covariates increase.^18^ The best LASSO parameter was identified through cross-validation using out-of-sample prediction with the least root mean squared error (appendix pp 24–25). To estimate CSMFs for each year in each country using the estimated parameters from the Bayesian LASSO model, we developed a prediction database with a complete time series of covariates from 2000 to 2019 for all 195 countries (appendix pp 8–10). To convert the CSMFs into cause-specific mortality rates and death counts, we used country estimates of all-cause mortality envelopes from UN-IGME (appendix pp 16–17).^4^, ^17^

For the 76 high mortality modelled (HMM) countries, we did a systematic review for verbal autopsy studies (appendix pp 11–13). In addition, we procured additional verbal autopsy studies through known investigator tracing (eg, the Million Death Study,^5^ the 2019 Pakistan Verbal Autopsy Social Autopsy Study [Bhutta ZA, University of Toronto, Totonto, ON, Canada, personal communication], Country wide Mortality Surveillance for Action [COMSA] in Mozambique,^22^ and a study from Madagascar^23^). We created separate databases for each of the four age-sex groups in which any studies providing information for the relevant group were included. Similar Bayesian LASSO models used for the LMM countries were applied to the HMM countries, with different covariates as inputs (appendix pp 8–10). In addition, on the basis of empirical evidence we only modelled malaria in individuals aged 5–14 years for HMM countries, and assumed zero malaria deaths in individuals aged 15 years and older. For high mortality country-years in which malaria incidence was zero according to the Global Malaria Program estimates,^24^ malaria deaths were also assumed to be zero. Considering the epidemiological evidence that deaths due to malaria in 5–14-year-olds do not exceed the proportions of deaths due to malaria in 1–59-month-olds,^25^, ^26^ we capped malaria CSMFs in this group at the 1–59-month level for the corresponding country-year and redistributed excess malaria deaths pro rata between the remaining causes post-hoc.^27^ The modelling process is summarised in the appendix (p 19).

### Single-cause estimates

In addition to modelled causes, we incorporated estimates of single causes with low burden or irregular patterns. These include HIV/AIDS, tuberculosis, measles, and crisis (collective violence, natural disasters, and infectious disease epidemics). We calculated UNAIDS estimated HIV/AIDS fractions on the basis of the envelopes used in Spectrum^28^ and applied them to the UN-IGME envelopes (the total number of deaths in each age-sex group in each country-year).^29^ We split HIV/AIDS out of the modelled fraction of other communicable, maternal, perinatal, and nutritional conditions (other CMPN). We split estimates of deaths due to measles produced by the WHO Immunization, Vaccines and Biologicals Department^30^ into endemic and epidemic components (appendix p 20). We adopted pulmonary and extrapulmonary tuberculosis estimates produced by the Global Tuberculosis Program^31^ and split them out of the modelled lower-respiratory infections and other CMPN fractions. We also used the median of the lower-respiratory tract infections and other CMPN fractions from high-quality vital registration data as the residual lower-respiratory tract infections and other CMPN, assuming high-quality vital registration countries have the minimum fractions of these causes. We then squeezed pro rata the single cause estimates of HIV/AIDS, endemic measles, extrapulmonary tuberculosis, and the residual other CMPN into the modelled other CMPN; pulmonary tuberculosis and residual lower-respiratory tract infections were added into the modelled lower-respiratory tract infections (appendix p 20). If for a given country single-cause estimates were not available for 2000–19, we assumed zero.

Crisis estimates were separately produced and redistributed into cause of death categories depending on the nature of the crisis (appendix p 21) and whether or not they were included in the UN-IGME envelope.^4^ To avoid excessive crisis deaths, for each country-year we capped the crisis fraction to the maximum fraction reported in the GHE^16^ for that country-year.

### China

For China, we relied on empirical data from the Disease Surveillance Points, a sample registration system for all ages covering 323·8 million people in China (Liu Y, Chu Y, Yeung D, et al, unpublished data). Specifically, we aggregated Disease Surveillance Points cause of death data to be consistent with the cause grouping used here and adjusted the empirical Disease Surveillance Points data for the complex sample design. We also applied the UN-IGME envelopes at the national level for 2004–19 to address under-counting^32^ and facilitate international comparison. We then extrapolated the 2004 CSMFs at the provincial levels for 2000–03 when empirical data were missing. For HIV/AIDS, we incorporated UNAIDS estimates following the same procedure described above. Details of the China national and subnational cause of death estimates are available elsewhere (Liu Y, Chu Y, Yeung D, et al, unpublished data).

### Uncertainty analysis

We assessed the uncertainty of the cause-specific mortality estimates based on the uncertainty of the model outputs, UN-IGME envelopes, and single-cause estimates. Uncertainty for modelled proportions were based on the posterior distribution of the Bayesian LASSO model parameters and random effect estimates.^18^ We accounted for uncertainty in the total number of deaths and mortality rates due to all causes by taking samples from the posterior distribution of the UN-IGME mortality envelopes.^4^ Uncertainty for the estimates of measles, tuberculosis, HIV/AIDS, and crisis were obtained by sampling from the corresponding uncertainty intervals on Monte Carlo simulations (appendix pp 17–18).

We followed the GATHER guidelines for global health estimates and included the GATHER checklist for transparency and replicability (appendix p 22). We did our analyses using the open-source statistical software R (version 4.1.1)^33^ and implemented the Bayesian modelling in JAGS (version 4.3.0),^34^ with wrapper functions from the R2jags package (version 0.7.1).^35^ These estimates have been reviewed and endorsed by representatives of WHO Member States through country consultation.

### Role of the funding source

The funder of the study had no role in study design, data collection, data analysis, data interpretation, or writing of the report.

## Results

In total, we used 12 642 cause of death input datapoints. They include 7710 datapoints from 67 countries for high-quality vital registration countries, 4241 empirical high-quality vital registration data points from 58 countries (excluding those with <15 deaths) for the LMM countries, and 691 verbal autopsy study datapoints from 29 countries for the HMM countries (appendix p 23). Not all LMM and HMM countries were represented in the input data due to data availability. In 2019, 1 480 227 deaths (95% uncertainty interval [UI] 1 426 105–1 598 461) occurred in 5–19-year-olds globally. 119 802 (8·1%) global deaths in 5–19-year-olds were reported in high-quality vital registration countries, 84 414 (5·7%) were reported in LMM countries, 1 221 418 (82·5%) were reported in HMM countries, and 54 593 (3·7%) were reported in China. The breakdown of the estimation inputs by age-sex groups and its comparison to outputs are presented in the appendix (p 23). The geographical distribution of input data is shown in the appendix (p 14). On the basis of the out-of-sample prediction with the SD of the random effect set at 0·07, the best LASSO penalty for each model is reported in the appendix (pp 24–25).

**Figure 1:**
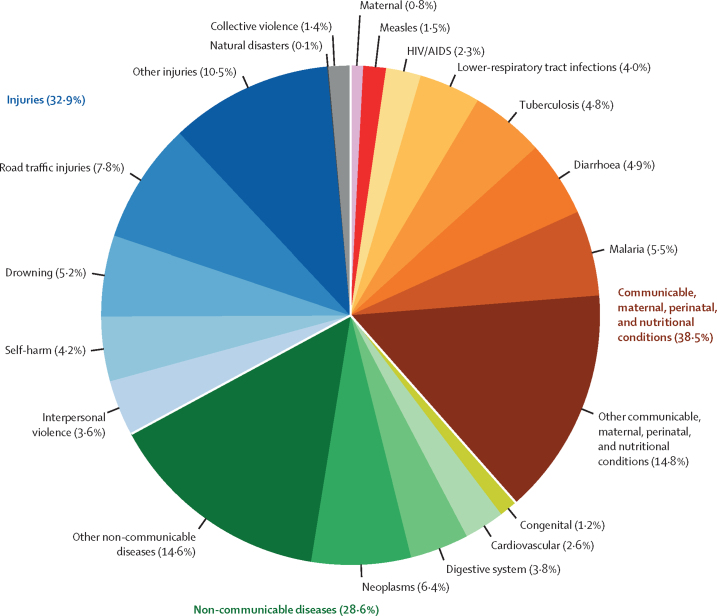
Global causes of death in 5–19-year-olds in 2019

Of the 1·48 million deaths in 5–19-year-olds, 569 930 (38·5%) deaths due to communicable, maternal, perinatal, and nutritional (CMPN) conditions were reported, 423 878 (28·6%) deaths due to non-communicable diseases were reported, and 486 403 (32·9%) deaths due to injuries were reported (figure 1). The leading specific causes of deaths in 2019 were road traffic injuries (115 843 [95% UI 110 672–125 054] deaths; 7·8% [7·5–8·1]), neoplasms (95 401 [90 744–104 812]; 6·4% [6·1–6·8]), malaria (81 516 [72 150–94 477]; 5·5% [4·9–6·2]), drowning (77 460 [72 474–85 952]; 5·2% [4·9–5·5]), and diarrhoea (72 679 [66 599–82 002]; 4·9% [4·5–5·3]; figure 1; table).

**Table:** Global overall and sex-age group specific causes of death in 2000 and 2019

	**Deaths in 2000**	**Deaths in 2019**
**All 5–19-year-olds**		
Road traffic injuries	162 355 (156 666–169 358)	115 843 (110 672–125 054)
Neoplasms	107 826 (103 578–112 917)	95 401 (90 744–104 812)
Malaria	116 250 (108 080–125 305)	81 516 (72 150–94 477)
Drowning	131 420 (123 226–140 348)	77 460 (72 474–85 952)
Diarrhoea	144 702 (136 371–154 976)	72 679 (66 599–82 002)
Tuberculosis	124 546 (104 041–128 195)	70 829 (55 460–74 740)
Self-harm	68 702 (65 555–72 518)	61 903 (57 290–67 651)
Lower-respiratory tract infections	82 132 (77 353–93 930)	58 883 (53 980–71 901)
Digestive system	101 716 (96 832–109 140)	56 323 (52 820–62 076)
Interpersonal violence	55 568 (53 408–58 265)	53 540 (50 355–57 928)
Cardiovascular	51 261 (47 872–55 491)	38 634 (35 273–43 091)
HIV/AIDS	34 351 (31 018–38 400)	33 527 (30 744–38 872)
Measles	57 732 (47 964–68 795)	21 759 (16 589–31 468)
Collective violence	18 226 (17 308–18 964)	21 034 (18 058–24 940)
Congenital	19 117 (17 390–21 270)	17 555 (15 862–19 462)
Maternal	18 056 (16 187–20 367)	12 167 (10 744–14 082)
Natural disasters	2270 (2149–2 398)	992 (926–1 063)
Other CMPN	317 199 (305 712–349 677)	218 570 (206 447–253 063)
Other non-communicable diseases	305 954 (295 723–324 203)	215 965 (206 872–234 160)
Other injuries	202 328 (193 615–212 492)	155 631 (143 573–176 297)
All cause	2 121 733 (2 080 030–2 194 627)	1 480 227 (1 426 105–1 598 461)
**5–9-year-olds**		
Diarrhoea	105 893 (99 981–112 931)	51 630 (47 206–56 235)
Malaria	81 802 (76 321–88 314)	49 934 (44 427–56 095)
Lower-respiratory tract infections	51 637 (47 003–60 188)	33 851 (29 448–41 005)
Drowning	53 273 (48 645–58 670)	33 086 (29 589–36 514)
Neoplasms	30 113 (28 469–31 995)	28 449 (25 901–30 925)
Road traffic injuries	41 132 (38 539–44 167)	27 989 (25 521–30 332)
Tuberculosis	36 710 (27 277–42 457)	22 369 (15 760–26 180)
Measles	57 737 (47 964–68 795)	21 764 (16 589–31 468)
Congenital	19 121 (17 390–21 270)	17 556 (15 862–19 462)
Digestive system	40 244 (37 176–43 844)	16 124 (14 255–18 161)
HIV/AIDS	19 173 (15 950–22 492)	8349 (7339–9270)
Collective violence	3890 (3772–4009)	5082 (4012–6097)
Natural disasters	618 (567–665)	331 (296–371)
Other CMPN	134 544 (124 684–151 495)	86 965 (77 417–96 835)
Other non-communicable diseases	115 353 (109 958–121 534)	67 718 (62 492–73 228)
Other injuries	79 095 (73 610–85 149)	45 778 (41 548–50 039)
All cause	870 327 (850 443–895 917)	516 971 (487 332–549 117)
**10–14-year-olds**		
Malaria	34 455 (28 378–41 164)	31 587 (23 940–43 116)
Neoplasms	29 796 (26 662–33 202)	26 820 (23 288–34 760)
Lower-respiratory tract infections	30 507 (26 560–36 943)	25 034 (21 127–34 916)
Drowning	48 477 (41 623–55 624)	23 610 (20 129–30 816)
Road traffic injuries	34 548 (30 618–39 376)	22 542 (19 431–28 636)
Diarrhoea	38 815 (32 487–46 474)	21 047 (16 751–28 938)
HIV/AIDS	7092 (5637–8618)	12 068 (9792–16 967)
Digestive system	22184 (19 438–27 815)	10 595 (8673–14 877)
Tuberculosis	11 575 (7206–15 214)	7823 (4737–10 010)
Collective violence	4890 (4436–5048)	5835 (3350–9273)
Natural disasters	746 (649–833)	298 (258–344)
Other CMPN	77 870 (69 730–92 122)	61 919 (52 009–84 811)
Other non-communicable diseases	92 261 (84 162–108 025)	58 396 (51 936–72 679)
Other injuries	57 247 (50 972–64 685)	60 572 (49 709–79 719)
All cause	490 461 (455 318–543 872)	368 151 (326 403–462 580)
**15–19-year-old females**		
Self-harm	36 989 (34 805–39 688)	32 646 (29 530–36 416)
Neoplasms	21 425 (19 655–23 587)	18 298 (16 233–21 042)
Cardiovascular	25 848 (23 167–29 399)	16 953 (14 823–19 879)
Road traffic injuries	19 697 (18 036–21 630)	16 559 (14 483–19 573)
Tuberculosis	35 496 (28 260–40 586)	15 369 (10 655–18 586)
Digestive system	20 920 (18 509–23 706)	12 937 (11 098–15 128)
Maternal	18 047 (16 187–20 367)	12 161 (10 744–14 082)
Interpersonal violence	9097 (8073–10 309)	10 437 (8 964–12 467)
HIV/AIDS	5045 (4264–5649)	5949 (5132–6931)
Drowning	6725 (5971–7656)	4531 (3915–5344)
Collective violence	3347 (3009–3694)	3315 (2936–3805)
Natural disasters	321 (286–360)	129 (107–153)
Other CMPN	53 887 (47 765–64 529)	28 460 (23 872–37 904)
Other non-communicable diseases	51 472 (48 164–55 557)	47 105 (42 787–52 882)
Other injuries	25 761 (23 196–29 006)	18 373 (15 677–21 614)
All cause	334 080 (325 183–346 764)	243 225 (227 530–263 597)
**15–19-year-old males**		
Road traffic injuries	66 979 (64 779–69 881)	48 757 (45 692–52 625)
Interpersonal violence	46 464 (44 609–48 813)	43 102 (40 579–46 263)
Self-harm	31 720 (29 935–33 922)	29 255 (27 258–32 024)
Tuberculosis	40 769 (31 917–45 086)	25 265 (18 457–28 629)
Neoplasms	26 487 (25 130–28 109)	21 834 (20 379–23 910)
Cardiovascular	25 407 (23 618–27 596)	21 686 (19 662–24 290)
Digestive system	18 364 (17 182–19 838)	16 665 (15 179–18 688)
Drowning	22 948 (21 615–24 510)	16 239 (14 785–18 005)
HIV/AIDS	3044 (2657–3585)	7154 (6246–8210)
Collective violence	6096 (5701–6519)	6795 (6109–7591)
Natural disasters	593 (541–649)	232 (200–266)
Other CMPN	50 912 (45 771–63 072)	41 237 (36 167–53 863)
Other non-communicable diseases	46 861 (44 638–49 359)	42 747 (39 705–46 396)
Other injuries	40 219 (38 045–42 820)	30 913 (28 396–34 100)
All cause	426 860 (416 713–441 895)	351 881 (331 858–377 736)

Data are number of deaths (95% uncertainty intervals). CMPN=communicable, maternal, perinatal, and nutritional conditions.

**Figure 2:**
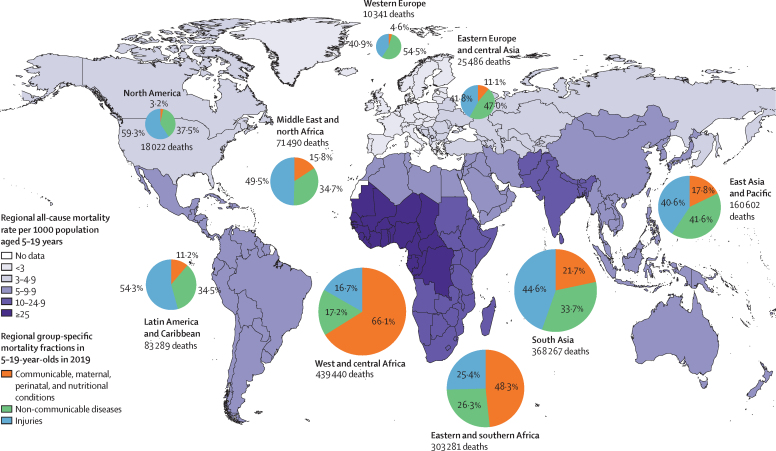
Regional all-cause mortality rates and group-specific mortality fractions in 5–19-year-olds in 2019

The mortality rates, number of deaths, and cause of death distribution in 5–19-year-olds also varied between regions in 2019 (figure 2). Of the nine regions presented, west and central Africa, south Asia, and eastern and southern Africa have the highest number of deaths in 5–19-year-olds. CMPN conditions are collectively dominant in west and central Africa and eastern and southern Africa, whereas injuries cause a higher proportion of deaths in other regions. The Middle East and north Africa was uniquely characterised by having collective violence as the leading cause of death. The leading cause of death in west and central Africa was malaria, whereas the leading cause of death in south Asia was road traffic injuries and HIV/AIDS in eastern and southern Africa (appendix pp 26–27).

Unique age patterns exist globally in the causes of deaths in 5–19-year-olds. The top three leading specific causes in each age-sex group in 2019 were diarrhoea, malaria, and lower-respiratory tract infections in 5–9-year-olds; malaria, neoplasms, and lower-respiratory tract infections in 10–14-year-olds; self-harm, neoplasms, and cardiovascular diseases in females aged 15–19 years; and road traffic injuries, interpersonal violence, and self-harm in males aged 15–19 (table).

**Figure 3:**
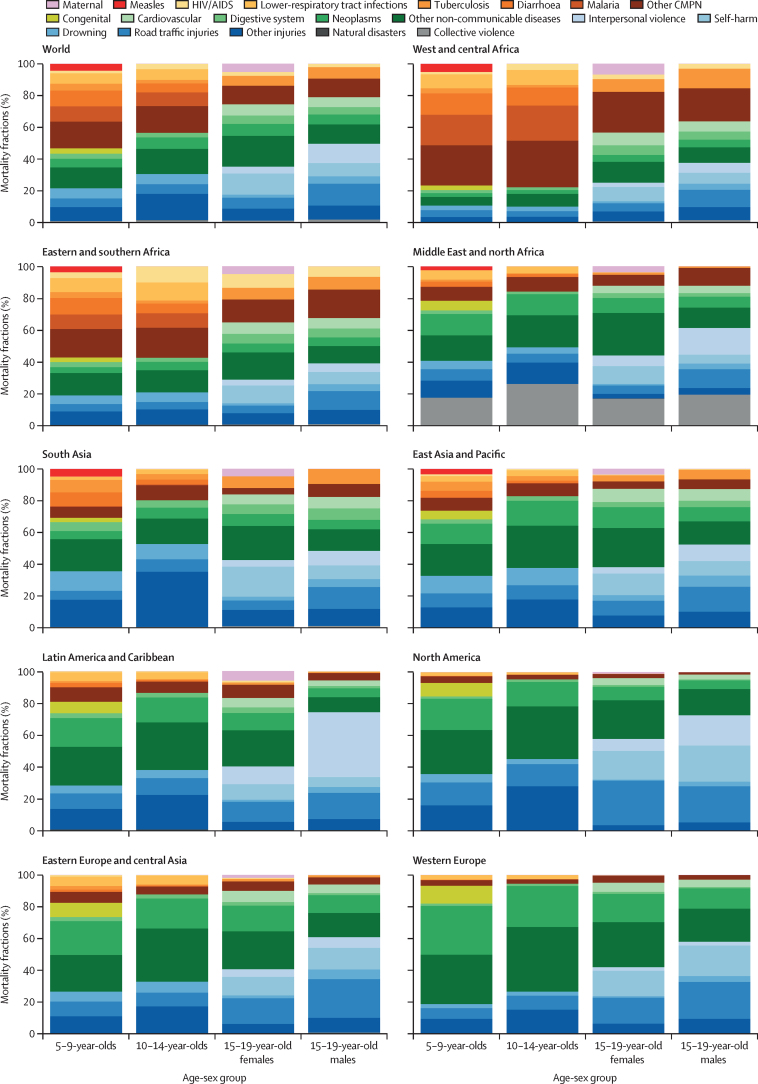
Global and regional causes of mortality fractions by age-sex group in 2019 Data used to create figure reported in the appendix (pp 28–31). CMPN=communicable, maternal, perinatal, and nutritional conditions.

The regional age-specific cause of death patterns are even more diverse (figure 3). As expected, overall the proportion of CMPN conditions reduced with age across all regions. However, in west and central Africa and eastern and southern Africa, CMPN conditions accounted for more than half of the age-specific deaths in 5–14-year-olds compared with less than a quarter in other regions. West and central Africa has a particularly large proportion of deaths in 5–14-year-olds due to malaria. HIV/AIDS causes a high proportion of deaths in 10–19-year-olds in eastern and southern Africa. Drowning is the leading cause of death in 5–9-year-olds in south Asia. In 15–19-year-old females, self-harm is the leading cause of death in south Asia, west and central Africa, eastern and southern Africa, and east Asia and Pacific, whereas collective violence is the leading cause of death in the Middle East and north Africa, and road traffic injuries and neoplasms are the leading specific causes in the remaining regions (North America, Latin America and the Caribbean, western Europe, and eastern Europe and central Asia). Across the nine regions, maternal causes contributed to the highest fraction of deaths in west and central Africa and Latin America and the Caribbean. In 15–19-year-old males, road traffic injuries were the leading specific cause in five regions (east Asia and Pacific, eastern and southern Africa, south Asia, western Europe, and eastern Europe and central Asia). In the same age-sex group, interpersonal violence is the most prevalent cause of death in Latin America and the Caribbean, self-harm—closely followed by road traffic injuries—is the most common in North America, and collective violence is the most common in the Middle East and north Africa (appendix pp 28–31).

At a national level, India, Nigeria, and the Demographic Republic of the Congo had the highest number of deaths in 5–19-year-olds in 2019 (appendix pp 41–42). In these settings, most deaths occurred in children aged 5–9 years. The leading causes in this age group were drowning, diarrhoea, and tuberculosis in India; malaria, diarrhoea, and lower-respiratory tract infections in Nigeria; and malaria, measles (2019 was an outbreak year), and diarrhoea in the Demographic Republic of the Congo (appendix pp 43–45).

**Figure 4:**
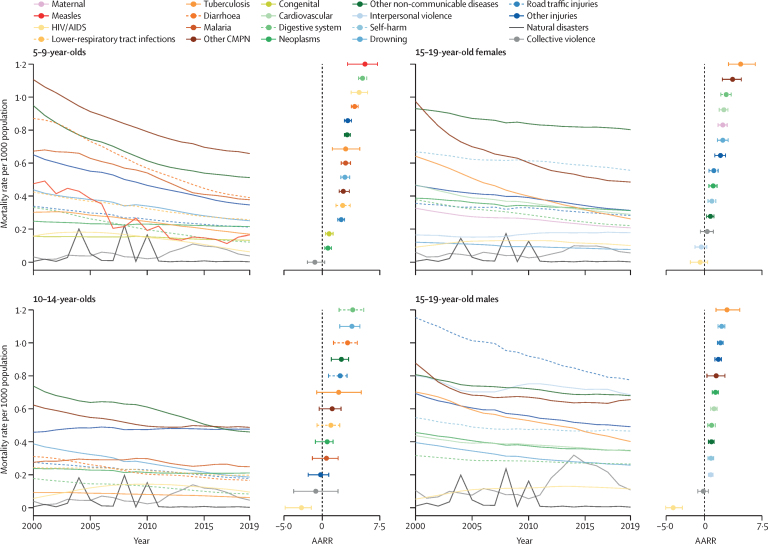
Global cause-specific mortality rates and AARR by age-sex group, 2000–19 Natural disasters not included in the calculation of AARR due to its idiosyncratic tendency. Horizontal lines in AARR panels denote 95% uncertainty intervals. AARR=annual average rate of reduction. CMPN=communicable, maternal, perinatal, and nutritional conditions.

From 2000 to 2019, the total number of deaths in 5–19-year-olds declined from 2·12 million (95% UI 2·08–2·19) to 1·48 million (1·43–1·60) globally. The decline is in general widespread across age, time, and cause of death (figure 4). In the four age-sex groups, the fastest decline was observed in measles for 5–9-year-olds at an annual average rate of reduction (AARR) of 5·57 [95% UI 3·30–7·21], followed by diseases of the digestive system in 5–14-year-olds (5·25 [4·74–5·81] for 5–9-year-olds and 3·97 [2·19–5·41] for 10–14-year-olds). In both 15–19-year-old females and males, tuberculosis had the largest AARR: 4·72 [3·14–6·63] for females and 2·94 [1·51–4·61] for males.

Deaths due to natural disasters were high in 2004 in Indonesia, Sri Lanka, Thailand, and India due to the Indian Ocean tsunami; in 2008 in China due to the Wenchuan earthquake; and 2010 in Haiti due to the Léogâne earthquake (appendix pp 36–37, 41, 43). Collective violence shows higher mortality in 2019 than 2000 in all age-sex groups except 15–19-year-old females. A higher mortality rate due to collective violence was estimated in 2011–19 compared with the previous decade with a peak in 2014 across all age-sex groups, primarily due to conflicts in Syria. HIV/AIDS mortality increased between 2000 and 2019; however, it was higher in 2010 than 2019.

Widespread decline in cause-specific mortality rates was also estimated across regions in 2000–19. For example, malaria mortality decreased quickly in 5–9-year-olds and gradually in 10–14-year-olds in west and central Africa (appendix p 32). Road traffic injuries mortality declined steadily in eastern Europe and central Asia, North America, and western Europe in 15–19-year-old males; however, interpersonal violence mortality had a flatter trend in the Middle East and north Africa in the same age-sex group (appendix pp 33–40). At a country level, India had the fastest decline in deaths due to diarrhoea in 5–9-year-olds (appendix p 43); Nigeria and the Demographic Republic of the Congo had the fastest decline in malaria mortality in the same age group (appendix pp 44–45).

## Discussion

In this study, we estimated the distribution of causes of death for 5–19-year-olds across age-sex groups, geographies, and time. Globally in 2019, the leading specific causes are road traffic injuries, neoplasms, malaria, drowning, and diarrhoea. However, the leading causes vary substantially across regions. The contribution of CMPN conditions declines with age, whereas the effect of injuries increases. The leading causes in each age-sex group globally are diarrhoea in 5–9-year-olds, malaria in 10–14-year-olds, self-harm in females aged 15–19 years, and road traffic injuries in males aged 15–19 years. Widespread declines in cause-specific mortality were estimated across age-sex groups and geographies. However, mortality due to collective violence increased in the past decade (2010–19) compared with the 2000s.

Road traffic injuries are the most common cause of death in all 5–19-year-olds, and in 15–19-year-old males specifically. The AARR of road traffic injuries was around 2% across all four age-sex groups. Compared with causes with high AARR (eg, measles in 5–9-years-olds) mortality rates due to road traffic injuries have declined at a moderate pace from 2000 to 2019. Due to increasing urbanisation and motorisation in LMICs, deaths due to road traffic injuries might experience a slower decline or even increase in the future.^36^ To accelerate the decline in road traffic injuries mortality, legislation (eg, drunk driving legislation coupled with enforcement, through policing or technology) could be effective.^36^

Neoplasms are the second leading cause of death in 10–14-year-olds of both sexes and 15–19-years-old females. The pace of neoplasm mortality decline has been slow across the age-sex groups. Some of the decline could be explained by advancement in access to early diagnosis and therapeutics (eg, for chronic myeloid leukaemia and non-Hodgkin lymphoma).^37^, ^38^, ^39^ But continued advancements in prevention, diagnosis, and therapeutics are needed for other neoplasms.^37^ Neoplasm mortality is probably underestimated due to undiagnosis and underdiagnosis in LMICs.^40^, ^41^

Drowning is a significant cause of death in individuals younger than 15 years and should be a particular focus for child and adolescent survival programmes in south Asia and east Asian and Pacific. Evidence of drowning intervention effectiveness targeting older children (>5 years old) is scarce,^42^ especially in LMICs, but the existing studies suggest the effectiveness of swimming instruction in school-aged children (4–12 years old).^43^, ^44^

Communicable diseases, such as malaria and diarrhoea are some of the most important specific causes of death in LMICs, particularly in west and central Africa, eastern and southern Africa, and south Asia. The reasons why diarrhoea remains high in 5–14-year-olds could be that in these settings, cholera and some other causes of diarrhoea (eg, enterotoxigenic *Escherichia coli*) occur throughout childhood and adulthood. Individuals aged 5–19 years, like those younger than 5 years, still die from insufficient use of therapeutic oral rehydration solutions, zinc, antibiotics, and immunisations (such as rotavirus vaccine for infants and cholera vaccine for older children). Integrated community and school case management and vaccinations could be effective in reducing malaria and diarrhoea mortality.^27^, ^45^

Collective violence is the only cause of death with a largely consistent increase across all four age-sex groups and the numbers are likely underestimated. This is primarily driven by conflicts in fragile and conflict-affected countries.^46^ These countries not only have direct and indirect conflict-related mortality, but also have prolonged disparities in health intervention coverage compared with countries in the same geographical region that have not been as severely affected by war.^47^, ^48^

We faced a number of challenges during the estimation process. First, a major data gap exists for cause of death in 5–19-year-olds. Of the 1·48 million deaths in 5–19-year-olds in 2019, 1·36 (91·1%) occurred in countries without high quality vital registration, and causes of 1·22 (82·5%) million deaths were estimated using a small number of verbal autopsy studies. Of the verbal autopsy studies, few are nationally representative; most are done at the subnational or community levels. All of which show deep disparities in data availability. Therefore, empirical data collection through national and subnational verbal autopsy studies and the set-up and maintenance of sample registration systems, similar to COMSA-Mozambique,^22^ should be a priority in LMICs. This will be particularly synergistic now that we have an estimation model allowing more influence from nationally representative verbal autopsy studies.

Compared with children younger than 5 years,^27^ cause of death in 5–19-year-olds is characterised by many more causes. As a result, we needed to model a higher number of causes (eg, 11 modelled causes in 15–19-year-old females compared with eight modelled causes in children aged 1–59 months). We chose to only estimate causes accounting for at least 3% of the age-sex-specific burden globally^14^, ^15^ to balance between the number of causes modelled, data availability, and model stability. As a result, we estimated large fractions of other CMPN, other non-communicable diseases, and other injuries. We also adopted single cause estimates and the limitations associated with those estimates are carried over.

Additionally, fewer studies and a smaller number of total deaths per study were available as model inputs in 5–19-year-olds compared with individuals younger than 5 years. For example, 520 cause of death input data points were available for 1–59-month-olds compared with 145 in 15–19-year-old males.^27^ The average number of total deaths per study were 1091 in neonates and 185 in 15–19-year-old males. This led to modelling instability and estimates are sensitive to modelling decisions. Partly due to data scarcity, our estimates are sensitive to the modelling group assignment. For selected countries with a probability of death from 5 to 19 around a cutoff of 10 per 1000 population, the estimates could be quite different if they were classified as HMM countries versus LMM countries. We attempted model averaging to address this uncommon discontinuity, but the results were too volatile to be plausible.^27^

We chose to produce estimates for 2000–19; we intentionally left out 2020 due to restricted knowledge on the direct and indirect effects of COVID-19. This highlights the acute and persistent data gap^49^ in child and adolescent mortality and cause of death.

Older children and adolescent survival need focused attention because cause of death in these age groups reflect changes in health risks with increasing age. Improving survival in these age groups requires multisectoral action from outside of the health domain—including education, transportation and road infrastructure, water and sanitation, and law enforcement, all of which have important, complementary roles in reducing mortality.^12^ From a life course perspective, we need to build on the survival gains achieved through focused investment on reducing mortality in children younger than 5 years mortality by extending those resources to prevent and treat injuries and non-communicable diseases in 5–19-year-olds. The global health community has become increasingly engaged in advocacy and actions to improve survival in this age group.^8^, ^50^ To translate these visions into actions, investments are needed to build country capacity in birth, death, and cause of death registration, sample registration, and surveys, so that life-saving interventions can be provided where they are most needed to improve survival across the life course for all children and adolescents.

## Data sharing

The source code, primary inputs and cause of death data collected and estimated are publicly available for research purposes on GitHub.

## Declaration of interests

LL, FV, DY, JP, and REB report grants from the Bill & Melinda Gates Foundation. All other authors declare no competing interests.
