# Supplementary material for: National, regional, and global causes of mortality in 5–19-year-olds from 2000 to 2019: a systematic analysis
Source: Lancet Glob Health. 2022 Feb 15;10(3):e337–47. doi: 10.1016/S2214-109X(21)00566-0 (PMC8864304; doi:10.1016/S2214-109X(21)00566-0)
Supplement: Supplementary appendix [file mmc1.pdf]

THE LANCET
Global Health

Supplementary appendix

This appendix formed part of the original submission and has been peer reviewed. We post it as supplied by the authors.

Supplement to: Liu L, Villavicencio F, Yeung D, et al. National, regional, and global causes of mortality in 5–19-year-olds from 2000 to 2019: a systematic analysis. *Lancet Glob Health* 2022; **10:** e337–47.

Table of contents

Webappendix 1. Country classification **3**

Webappendix 2. Cause categorization and ICD codes **7**

Webappendix 3. Model input data procurement and preparation **8**

Webappendix 3.1 Covariates and their preparation 8

Webappendix 3.2 COD model inputs preparation 11

Webappendix 3.3 Input data map 14

Webappendix 4. Additional details of modeling and estimation **15**

Webappendix 4.1 The Bayesian LASSO 15

Webappendix 4.2 Estimation of the sex-specific deaths and rates for 15-19 16

Webappendix 4.3 Point estimates and uncertainty 17

Webappendix 4.4 Transparency and replicability 18

Webappendix 5. Modeling and estimation strategies **19**

Webappendix 6. Additional details on single cause estimates **20**

Webappendix 6.1 Splitting measles into endemic and epidemic 20

Webappendix 6.2 The process of squeezing extrapulmonary TB, HIV/AIDS and endemic measles into Other CMPN 20

Webappendix 6.3 Crisis estimates 21

Webappendix 7. The GATHER checklist **22**

Webappendix 8. Inputs and outputs by estimation methods **23**

Webappendix 9. Model selection process **24**

Webappendix 9.1 Cross-validation for low-mortality model (LMM) 24

Webappendix 9.2 Cross-validation for high-mortality model (HMM) 25

Webappendix 10. Global and regional cause-specific mortality estimates by age-sex group, 2000-2019 ... 26

Webappendix 10.1 Data files with global and regional cause-specific mortality estimates ... 26

Webappendix 10.2 Global and regional leading causes of death 5-19 years in 2019 ... 27

Webappendix 10.3 Global and regional causes of mortality fractions by age-sex group in 2019 ... 28

Webappendix 10.4 Regional cause-specific mortality rates by age-sex group, time trends 2000-2019 ... 32

Webappendix 11. National cause-specific mortality estimates by age-sex group, 2000-2019 ... 41

Webappendix 11.1 Data files with national cause-specific mortality estimates ... 41

Webappendix 11.2 Countries with the highest number of deaths in 2019 ... 42

Webappendix 11.3 Cause-specific mortality rates by age-sex group in India, Nigeria and Democratic Republic of Congo, time trends 2000-2019 ... 43

References ... 46

Webappendix 1. Country classification

High-quality VR countries were based on WHO criteria on the quality, completeness of vital registration (VR) data for ages 15 and above, and usability.[1]

We used a 10/1000 mortality rate in 2010 from 5 to 20 years of age from UN-IGME[2] as the threshold that separates low and high mortality model countries. The mortality rate between ages 5 and 20 years, denoted as ${}_{15}q_5$, is defined as the probability of dying between exact ages 5 and 20, expressed per 1,000 population aged 5.[2] We identified percentiles aligned with those used for the country classification for children under-five:[3]10/1000 is the 70 percentile of ${}_{15}q_5$ for all countries, and the 50 percentile of ${}_{15}q_5$ for all countries excluding those with high-quality VR data in 2010.

Below is the list of the 195 countries for which we provide cause-of-death estimates. The table also includes their regional classification, model category, and whether they have been flagged as a fragile state.[4] These are the same 195 countries for which UN-IGME provides estimates, which corresponds to the 194 WHO member states plus the West Bank and Gaza Strip.[2]

Table S1. List of the 195 countries and their classification

ISO3	WHO name	Region	Model category	Fragile state
AFG	Afghanistan	South Asia	High mortality model (HMM)	YES
AGO	Angola	Eastern and Southern Africa	High mortality model (HMM)	NO
ALB	Albania	Eastern Europe and central Asia	Low mortality model (LMM)	NO
AND	Andorra	Western Europe	Low mortality model (LMM)	NO
ARE	United Arab Emirates	Middle East and North Africa	Low mortality model (LMM)	NO
ARG	Argentina	Latin America and Caribbean	High-quality VR	NO
ARM	Armenia	Eastern Europe and central Asia	High-quality VR	NO
ATG	Antigua and Barbuda	Latin America and Caribbean	High-quality VR	NO
AUS	Australia	East Asia and Pacific	High-quality VR	NO
AUT	Austria	Western Europe	High-quality VR	NO
AZE	Azerbaijan	Eastern Europe and central Asia	Low mortality model (LMM)	NO
BDI	Burundi	Eastern and Southern Africa	High mortality model (HMM)	YES
BEL	Belgium	Western Europe	High-quality VR	NO
BEN	Benin	West and central Africa	High mortality model (HMM)	NO
BFA	Burkina Faso	West and central Africa	High mortality model (HMM)	YES
BGD	Bangladesh	South Asia	High mortality model (HMM)	NO
BGR	Bulgaria	Eastern Europe and central Asia	High-quality VR	NO
BHR	Bahrain	Middle East and North Africa	Low mortality model (LMM)	NO
BHS	Bahamas	Latin America and Caribbean	Low mortality model (LMM)	NO
BIH	Bosnia and Herzegovina	Eastern Europe and central Asia	Low mortality model (LMM)	NO
BLR	Belarus	Eastern Europe and central Asia	Low mortality model (LMM)	NO
BLZ	Belize	Latin America and Caribbean	Low mortality model (LMM)	NO
BOL	Bolivia (Plurinational State of)	Latin America and Caribbean	High mortality model (HMM)	NO
BRA	Brazil	Latin America and Caribbean	High-quality VR	NO
BRB	Barbados	Latin America and Caribbean	High-quality VR	NO
BRN	Brunei Darussalam	East Asia and Pacific	High-quality VR	NO
BTN	Bhutan	South Asia	High mortality model (HMM)	NO
BWA	Botswana	Eastern and Southern Africa	High mortality model (HMM)	NO
CAF	Central African Republic	West and central Africa	High mortality model (HMM)	YES
CAN	Canada	North America	High-quality VR	NO
CHE	Switzerland	Western Europe	High-quality VR	NO
CHL	Chile	Latin America and Caribbean	High-quality VR	NO
CHN	China	East Asia and Pacific	China DSP	NO
CIV	Côte d'Ivoire	West and central Africa	High mortality model (HMM)	NO

ISO3	WHO name	Region	Model category	Fragile state
CMR	Cameroon	West and central Africa	High mortality model (HMM)	YES
COD	Democratic Republic of the Congo	West and central Africa	High mortality model (HMM)	YES
COG	Congo	West and central Africa	High mortality model (HMM)	YES
COK	Cook Islands	East Asia and Pacific	Low mortality model (LMM)	NO
COL	Colombia	Latin America and Caribbean	High-quality VR	NO
COM	Comoros	Eastern and Southern Africa	High mortality model (HMM)	YES
CPV	Cabo Verde	West and central Africa	Low mortality model (LMM)	NO
CRI	Costa Rica	Latin America and Caribbean	High-quality VR	NO
CUB	Cuba	Latin America and Caribbean	High-quality VR	NO
CYP	Cyprus	Western Europe	High-quality VR	NO
CZE	Czechia	Western Europe	High-quality VR	NO
DEU	Germany	Western Europe	High-quality VR	NO
DJI	Djibouti	Eastern and Southern Africa	High mortality model (HMM)	NO
DMA	Dominica	Latin America and Caribbean	Low mortality model (LMM)	NO
DNK	Denmark	Western Europe	High-quality VR	NO
DOM	Dominican Republic	Latin America and Caribbean	Low mortality model (LMM)	NO
DZA	Algeria	Middle East and North Africa	Low mortality model (LMM)	NO
ECU	Ecuador	Latin America and Caribbean	High-quality VR	NO
EGY	Egypt	Middle East and North Africa	Low mortality model (LMM)	NO
ERI	Eritrea	Eastern and Southern Africa	High mortality model (HMM)	YES
ESP	Spain	Western Europe	High-quality VR	NO
EST	Estonia	Western Europe	High-quality VR	NO
ETH	Ethiopia	Eastern and Southern Africa	High mortality model (HMM)	NO
FIN	Finland	Western Europe	High-quality VR	NO
FJI	Fiji	East Asia and Pacific	High mortality model (HMM)	NO
FRA	France	Western Europe	High-quality VR	NO
FSM	Micronesia (Federated States of)	East Asia and Pacific	High mortality model (HMM)	YES
GAB	Gabon	West and central Africa	High mortality model (HMM)	NO
GBR	United Kingdom of Great Britain and Northern Ireland	Western Europe	High-quality VR	NO
GEO	Georgia	Eastern Europe and central Asia	Low mortality model (LMM)	NO
GHA	Ghana	West and central Africa	High mortality model (HMM)	NO
GIN	Guinea	West and central Africa	High mortality model (HMM)	NO
GMB	Gambia	West and central Africa	High mortality model (HMM)	YES
GNB	Guinea-Bissau	West and central Africa	High mortality model (HMM)	YES
GNQ	Equatorial Guinea	West and central Africa	High mortality model (HMM)	NO
GRC	Greece	Western Europe	High-quality VR	NO
GRD	Grenada	Latin America and Caribbean	High-quality VR	NO
GTM	Guatemala	Latin America and Caribbean	High mortality model (HMM)	NO
GUY	Guyana	Latin America and Caribbean	High-quality VR	NO
HND	Honduras	Latin America and Caribbean	Low mortality model (LMM)	NO
HRV	Croatia	Eastern Europe and central Asia	High-quality VR	NO
HTI	Haiti	Latin America and Caribbean	High mortality model (HMM)	YES
HUN	Hungary	Western Europe	High-quality VR	NO
IDN	Indonesia	East Asia and Pacific	High mortality model (HMM)	NO
IND	India	South Asia	High mortality model (HMM)	NO
IRL	Ireland	Western Europe	High-quality VR	NO
IRN	Iran (Islamic Republic of)	Middle East and North Africa	High mortality model (HMM)	NO
IRQ	Iraq	Middle East and North Africa	Low mortality model (LMM)	YES
ISL	Iceland	Western Europe	High-quality VR	NO
ISR	Israel	Middle East and North Africa	High-quality VR	NO
ITA	Italy	Western Europe	High-quality VR	NO
JAM	Jamaica	Latin America and Caribbean	Low mortality model (LMM)	NO
JOR	Jordan	Middle East and North Africa	Low mortality model (LMM)	NO
JPN	Japan	East Asia and Pacific	High-quality VR	NO
KAZ	Kazakhstan	Eastern Europe and central Asia	Low mortality model (LMM)	NO
KEN	Kenya	Eastern and Southern Africa	High mortality model (HMM)	NO
KGZ	Kyrgyzstan	Eastern Europe and central Asia	Low mortality model (LMM)	NO

ISO3	WHO name	Region	Model category	Fragile state
KHM	Cambodia	East Asia and Pacific	High mortality model (HMM)	NO
KIR	Kiribati	East Asia and Pacific	High mortality model (HMM)	YES
KNA	Saint Kitts and Nevis	Latin America and Caribbean	Low mortality model (LMM)	NO
KOR	Republic of Korea	East Asia and Pacific	High-quality VR	NO
KWT	Kuwait	Middle East and North Africa	Low mortality model (LMM)	NO
LAO	Lao People's Democratic Republic	East Asia and Pacific	High mortality model (HMM)	YES
LBN	Lebanon	Middle East and North Africa	Low mortality model (LMM)	YES
LBR	Liberia	West and central Africa	High mortality model (HMM)	YES
LBY	Libya	Middle East and North Africa	Low mortality model (LMM)	YES
LCA	Saint Lucia	Latin America and Caribbean	High-quality VR	NO
LKA	Sri Lanka	South Asia	Low mortality model (LMM)	NO
LSO	Lesotho	Eastern and Southern Africa	High mortality model (HMM)	NO
LTU	Lithuania	Western Europe	High-quality VR	NO
LUX	Luxembourg	Western Europe	High-quality VR	NO
LVA	Latvia	Western Europe	High-quality VR	NO
MAR	Morocco	Middle East and North Africa	Low mortality model (LMM)	NO
MCO	Monaco	Western Europe	Low mortality model (LMM)	NO
MDA	Republic of Moldova	Eastern Europe and central Asia	High-quality VR	NO
MDG	Madagascar	Eastern and Southern Africa	High mortality model (HMM)	NO
MDV	Maldives	South Asia	Low mortality model (LMM)	NO
MEX	Mexico	Latin America and Caribbean	High-quality VR	NO
MHL	Marshall Islands	East Asia and Pacific	High mortality model (HMM)	YES
MKD	Republic of North Macedonia	Eastern Europe and central Asia	High-quality VR	NO
MLI	Mali	West and central Africa	High mortality model (HMM)	YES
MLT	Malta	Western Europe	High-quality VR	NO
MMR	Myanmar	East Asia and Pacific	High mortality model (HMM)	YES
MNE	Montenegro	Eastern Europe and central Asia	Low mortality model (LMM)	NO
MNG	Mongolia	East Asia and Pacific	High mortality model (HMM)	NO
MOZ	Mozambique	Eastern and Southern Africa	High mortality model (HMM)	YES
MRT	Mauritania	West and central Africa	High mortality model (HMM)	NO
MUS	Mauritius	Eastern and Southern Africa	High-quality VR	NO
MWI	Malawi	Eastern and Southern Africa	High mortality model (HMM)	NO
MYS	Malaysia	East Asia and Pacific	Low mortality model (LMM)	NO
NAM	Namibia	Eastern and Southern Africa	High mortality model (HMM)	NO
NER	Niger	West and central Africa	High mortality model (HMM)	YES
NGA	Nigeria	West and central Africa	High mortality model (HMM)	YES
NIC	Nicaragua	Latin America and Caribbean	High-quality VR	NO
NLD	Netherlands	Western Europe	High-quality VR	NO
NOR	Norway	Western Europe	High-quality VR	NO
NPL	Nepal	South Asia	High mortality model (HMM)	NO
NRU	Nauru	East Asia and Pacific	High mortality model (HMM)	NO
NUI	Niue	East Asia and Pacific	High mortality model (HMM)	NO
NZL	New Zealand	East Asia and Pacific	High-quality VR	NO
OMN	Oman	Middle East and North Africa	Low mortality model (LMM)	NO
PAK	Pakistan	South Asia	High mortality model (HMM)	NO
PAN	Panama	Latin America and Caribbean	High-quality VR	NO
PER	Peru	Latin America and Caribbean	High-quality VR	NO
PHL	Philippines	East Asia and Pacific	High-quality VR	NO
PLW	Palau	East Asia and Pacific	High mortality model (HMM)	NO
PNG	Papua New Guinea	East Asia and Pacific	High mortality model (HMM)	YES
POL	Poland	Western Europe	High-quality VR	NO
PRK	Democratic People's Republic of Korea	East Asia and Pacific	High mortality model (HMM)	NO
PRT	Portugal	Western Europe	High-quality VR	NO
PRY	Paraguay	Latin America and Caribbean	High-quality VR	NO
PSE	West Bank and Gaza Strip	Middle East and North Africa	Low mortality model (LMM)	YES
QAT	Qatar	Middle East and North Africa	Low mortality model (LMM)	NO
ROU	Romania	Eastern Europe and central Asia	High-quality VR	NO
RUS	Russian Federation	Eastern Europe and central Asia	Low mortality model (LMM)	NO

ISO3	WHO name	Region	Model category	Fragile state
RWA	Rwanda	Eastern and Southern Africa	High mortality model (HMM)	NO
SAU	Saudi Arabia	Middle East and North Africa	Low mortality model (LMM)	NO
SDN	Sudan	Eastern and Southern Africa	High mortality model (HMM)	YES
SEN	Senegal	West and central Africa	High mortality model (HMM)	NO
SGP	Singapore	East Asia and Pacific	High-quality VR	NO
SLB	Solomon Islands	East Asia and Pacific	High mortality model (HMM)	YES
SLE	Sierra Leone	West and central Africa	High mortality model (HMM)	NO
SLV	El Salvador	Latin America and Caribbean	High-quality VR	NO
SMR	San Marino	Western Europe	Low mortality model (LMM)	NO
SOM	Somalia	Eastern and Southern Africa	High mortality model (HMM)	YES
SRB	Serbia	Eastern Europe and central Asia	High-quality VR	NO
SSD	South Sudan	Eastern and Southern Africa	High mortality model (HMM)	YES
STP	Sao Tome and Principe	West and central Africa	High mortality model (HMM)	NO
SUR	Suriname	Latin America and Caribbean	Low mortality model (LMM)	NO
SVK	Slovakia	Western Europe	High-quality VR	NO
SVN	Slovenia	Western Europe	High-quality VR	NO
SWE	Sweden	Western Europe	High-quality VR	NO
SWZ	Eswatini	Eastern and Southern Africa	High mortality model (HMM)	NO
SYC	Seychelles	Eastern and Southern Africa	Low mortality model (LMM)	NO
SYR	Syrian Arab Republic	Middle East and North Africa	Low mortality model (LMM)	YES
TCD	Chad	West and central Africa	High mortality model (HMM)	YES
TGO	Togo	West and central Africa	High mortality model (HMM)	NO
THA	Thailand	East Asia and Pacific	High mortality model (HMM)	NO
TJK	Tajikistan	Eastern Europe and central Asia	Low mortality model (LMM)	NO
TKM	Turkmenistan	Eastern Europe and central Asia	Low mortality model (LMM)	NO
TLS	Timor-Leste	East Asia and Pacific	High mortality model (HMM)	YES
TON	Tonga	East Asia and Pacific	Low mortality model (LMM)	NO
TTO	Trinidad and Tobago	Latin America and Caribbean	Low mortality model (LMM)	NO
TUN	Tunisia	Middle East and North Africa	Low mortality model (LMM)	NO
TUR	Turkey	Eastern Europe and central Asia	Low mortality model (LMM)	NO
TUV	Tuvalu	East Asia and Pacific	High mortality model (HMM)	YES
TZA	United Republic of Tanzania	Eastern and Southern Africa	High mortality model (HMM)	NO
UGA	Uganda	Eastern and Southern Africa	High mortality model (HMM)	NO
UKR	Ukraine	Eastern Europe and central Asia	Low mortality model (LMM)	NO
URY	Uruguay	Latin America and Caribbean	High-quality VR	NO
USA	United States of America	North America	High-quality VR	NO
UZB	Uzbekistan	Eastern Europe and central Asia	Low mortality model (LMM)	NO
VCT	Saint Vincent and the Grenadines	Latin America and Caribbean	High-quality VR	NO
VEN	Venezuela (Bolivarian Republic of)	Latin America and Caribbean	High-quality VR	YES
VNM	Viet Nam	East Asia and Pacific	Low mortality model (LMM)	NO
VUT	Vanuatu	East Asia and Pacific	High mortality model (HMM)	NO
WSM	Samoa	East Asia and Pacific	Low mortality model (LMM)	NO
YEM	Yemen	Middle East and North Africa	High mortality model (HMM)	YES
ZAF	South Africa	Eastern and Southern Africa	High mortality model (HMM)	NO
ZMB	Zambia	Eastern and Southern Africa	High mortality model (HMM)	NO
ZWE	Zimbabwe	Eastern and Southern Africa	High mortality model (HMM)	YES

Webappendix 2. Cause categorization and ICD codes

Specific causes of death (COD) that made up at least 3% of global deaths in 2016 among any of the adolescent 5-year-age-and-sex groups according to the existing estimates[4,5] were considered for modeling. The final causes were influenced by model stability, which was driven by the frequency and magnitude of the cause fractions in the empirical data. Remaining causes were grouped into the respective "other" categories (Other communicable, maternal, perinatal and nutritional conditions – Other CMPN; Other non-communicable diseases – Other NCD; and Other injuries).

Table S2. Cause list and the ICD code mapping

Cause name	ICD-10
Communicable, maternal, perinatal, and nutritional conditions	A00-B99, D50-D53, D64·9, E00-E02, E40-E46, E50-E68, G00, G03-G04, H65-H66, J00-J22, N70-N73, O00-O99, P00-P96 (except P23, P37·3, P37·4), U04
HIV/AIDS	B20-B24
Diarrheal	A00, A01, A03, A04, A06-A09
Measles	B05
Malaria	B50-B54
Lower Respiratory Infections	J09-J22, P23, U04
Tuberculosis	A15-A19, B90
Maternal causes	O00-O99
Other CMPN	Remainder of communicable, maternal, perinatal, and nutritional conditions
Non-communicable diseases	C00-C97, D00-D48, D55-D89 (except D64.9), E03-E07, E10-E34, E65-E88, F01-F99, G06-G98, H00-H61, H68-H93, I00-I99, J30-J98, K00-K92, L00-L98, M00-M99, N00-N64, N75-N98, Q00-Q99
Congenital anomalies	Q00-Q99
Neoplasms	C00-D48
Cardiovascular disease	I00-I99
Diseases of the digestive system	K20-K92
Other NCD	Remainder of non-communicable diseases
Injuries	V01-Y09, Y35-Y36, Y40-Y86, Y88-Y89, Y871
Road traffic injuries	V01-V04, V06, V09-V80, V87, V89, V99
Drownings	W65-W74
Natural disaster	X30-X39
Interpersonal violence	X85-Y09, Y871
Collective violence: legal intervention	Y35
Collective violence: war	Y36
Self-harm	X60-X84, Y871
Other injuries	Remainder of injuries
Ill-defined	R00-R99, Y10-Y34, Y872

Webappendix 3. Model input data procurement and preparation

Webappendix 3.1 Covariates and their preparation

Each covariate had at least 200 empirical country data points between 1980 and 2017, and covariates with high age-sex resolution were prioritized. Covariates were matched to the study population using the following hierarchy: location, year, age, and sex.

For covariates, empirical data was first taken directly from their source without adjustments. A complete time-series for each country was then generated using linear interpolation between existing empirical data points, extrapolation assuming a flat trend for years preceding or succeeding the last available empirical data points, and imputation based on region and country's lag distributed GDP where there was no empirical data. These time series were used for country-years in the input database that did not have subnational covariate data. A smoothed times series was also generated using a 7-year average for model prediction.

Table S3.1. List of Covariates

Covariate	Description	Source	Included in final model
Alcohol consumption	Alcohol consumption per capita (in liters of pure alcohol)	World Health Organization Global Health Observatory (WHO GHO)	10-14 HMM<br>10-14 LMM<br>15-19 Males HMM<br>15-19 Males LMM<br>15-19 Females HMM<br>15-19 Females LMM
Depression	Prevalence of Depressive disorders (%)	Institute for Health Metrics and Evaluation Global Burden of Disease	10-14 HMM<br>15-19 Males HMM<br>15-19 Males LMM<br>15-19 Females HMM
ORS	Oral Rehydration Salts (%)	USAID Demographic Health Surveys (DHS) and UNICEF Multiple Indicator Cluster Surveys (MICS)	10-14 HMM
DTP3 vaccine	Diphtheria-tetanus-pertussis vaccine	DHS and MICS	5-9 HMM<br>10-14 HMM
Institutional delivery	Place of delivery: Health facility	DHS	15-19 Females HMM<br>15-19 Females LMM
LBW	Low birth weight	The World Bank	5-9 HMM
Adolescent birth rate	Age-specific fertility rates (births per 1,000 women)	United Nations Population Division World Population Prospects (UNDP WPP)	15-19 Females LMM
Age at first sexual intercourse 15	Percentage of people who had first sexual intercourse by exact age 15)	DHS	10-14 HMM<br>10-14 LMM<br>15-19 Males LMM<br>15-19 Females HMM
Marriage	Mean age at marriage	UNDP World Marriage Data	15-19 Males HMM<br>15-19 Males LMM<br>15-19 Females LMM
Early childbearing	Average age of mothers at the birth of their children	UN WPP	15-19 Females HMM<br>15-19 Females LMM

**Covariate**	**Description**	**Source**	**Included in final model**
Unmet need for family planning	Women unmet need for family planning (%)	UNPD World Contraceptive Use	15-19 Females HMM
Corruption	Perceived levels of public sector corruption according to experts and businesspeople	Transparency International Corruption Perceptions Index	5-9 HMM<br>5-9 LMM<br>10-14 HMM<br>10-14 LMM<br>15-19 Males HMM<br>15-19 Males LMM
Mean years of schooling	Average Years of Total Schooling	Barro-Lee Educational Attainment Dataset	5-9 HMM<br>5-9 LMM<br>10-14 LMM<br>15-19 Males LMM<br>15-19 Females LMM
Labor force participation	Labor force participation rate (%)	The World Bank	15-19 Males HMM
Unemployment	unemployment % of total labor force	The World Bank	15-19 Males HMM
Youth NEET	Youth NEET (not in employment, education, or training) rate (%)	International Labour Organization	5-9 HMM<br>5-9 LMM<br>10-14 LMM<br>15-19 Males LMM<br>15-19 Females LMM
Literacy	Youth literacy rate, population 15-24 years	United Nations Educational, Scientific and Cultural Organization Institute for Statistics	15-19 Males HMM<br>15-19 Females HMM
People living in lowest wealth quintile	Population in the lowest wealth quintile (%)	DHS	5-9 HMM<br>5-9 LMM<br>10-14 HMM<br>10-14 LMM<br>15-19 Males HMM<br>15-19 Males LMM<br>15-19 Females HMM<br>15-19 Females LMM
Height	Mean height at age 18 (cm)	NCD Risk Factor Collaboration (NCD RisC)	5-9 HMM
Underweight	Prevalence of WAZ < 2 SD	Stevens *et al.* (2012)[6]	5-9 LMM<br>10-14 LMM
Obese	Obese (BMI>+2 standard deviation above the median)	NCD RisC	5-9 LMM<br>10-14 LMM<br>15-19 Males LMM<br>15-19 Females LMM
Thinness	Thinness (BMI<-2 standard deviation below the median)	NCD RisC	5-9 HMM<br>5-9 LMM<br>10-14 HMM<br>10-14 LMM<br>15-19 Males LMM<br>15-19 Females HMM<br>15-19 Females LMM
PfPR	P. falciparum parasite rate	Oxford Malaria Atlas Project	5-9 HMM<br>10-14 HMM

Covariate	Description	Source	Included in final model
Air pollution	PM 2.5 aerosol mass	NASA Merra-2[7]	5-9 HMM<br>5-9 LMM<br>10-14 HMM<br>10-14 LMM<br>15-19 Males LMM<br>15-19 Females LMM
Urban	Urban population %	UNDP World Urbanization Prospects	5-9 HMM<br>5-9 LMM<br>10-14 HMM<br>10-14 LMM<br>15-19 Males HMM<br>15-19 Males LMM<br>15-19 Females HMM<br>15-19 Females LMM
GINI	GINI Index	The World Bank	5-9 HMM<br>10-14 HMM<br>15-19 Males HMM<br>15-19 Females HMM
Population under 5	Proportion of population aged 0-4 years old	UNDP WPP	5-9 LMM
Population male 15-29	Proportion of population aged 15–30 years old and male	UNDP WPP	15-19 Males LMM
MR5to19	5-19 Mortality rate	UN-IGME	5-9 HMM<br>10-14 HMM<br>15-19 Females HMM<br>15-19 Females LMM
Year			5-9 LMM<br>10-14 LMM<br>15-19 Males HMM<br>15-19 Males LMM

Webappendix 3.2 COD model inputs preparation

Systematic review for verbal autopsy studies

We conducted a systematic review for verbal autopsy studies published between January 1, 1980, and December 31, 2017 to be used as model inputs. We used search terms related to COD, ages 5-19, and HMM countries in major search engines covering global health and epidemiology journals (Yeung D, Feng Y, Hong J, *et al.*; unpublished data). Articles were screened with pre-set inclusion and exclusion criteria and abstracted by two independent researchers using DistillerSR software.[8] We started with 55,471 articles and eventually included 62 in the final analysis.

Search items for systematic review

We searched on Pubmed, Scopus, Embase, Web of Science, Global Health Index Medicus, Global Health OVID, IndMed, PAHO, Popline, and Cochrane for studies published between January 1, 1980 and December 31, 2017, using the following search terms:

(("Adolescent" OR "adolescents" OR "adolescence" OR "teen" OR "teens" OR "teenager" OR "teenagers" OR "youth" OR "youths" OR "young adult" OR "young adults" OR "young person" OR "young people" OR "preteen" OR "preteens") OR
(("child" OR "children") AND
("6 years" OR "7 years" OR "8 years" OR "9 years" OR "10 years" OR "11 years" OR "12 years" OR "13 years" OR "14 years" OR "15 years" OR "school age" OR "age 6" OR "ages 6" OR "age 7" OR "ages 7" OR "age 8" OR "ages 8" OR "age 9" OR "ages 9" OR "age 10" OR "ages 10" OR "age 11" OR "ages 11" OR "age 12" OR "ages 12" OR "age 13" OR "ages 13" OR "age 14" OR "ages 14" OR "age 15" OR "ages 15"))) AND

(("Cause of death" OR “causes of death”) OR
(("cause" OR "causes" OR "autopsy" OR "autopsies" OR "disease burden" OR "survey" OR "surveys" OR "surveillance" OR "register" OR "registers" OR "registration" OR "vital statistics" OR "report" OR "reports") AND
("Mortality" OR "Mortalities" OR "fatal" OR "fatality" OR "fatalities" OR "death" OR "deaths"))) AND

("Afghanistan" OR "Algeria" OR "Angola" OR "Azerbaijan" OR "Bangladesh" OR "Benin" OR "Bhutan" OR "Bolivia" OR "Botswana" OR "Burkina Faso" OR "Burundi" OR "Cambodia" OR "Cameroon" OR "Central African Republic" OR "Chad" OR "China" OR "Comoros" OR "Congo" OR "Cote d'Ivoire" OR "Democratic People's Republic of Korea" OR "Congo" OR "Djibouti" OR "Dominican Republic" OR "Equatorial Guinea" OR "Eritrea" OR "Ethiopia" OR "Gabon" OR "Gambia" OR "Ghana" OR "Guatemala" OR "Guinea" OR "Guinea-Bissau" OR "Haiti" OR "India" OR "Indonesia" OR "Iran" OR "Iraq" OR "Kazakhstan" OR "Kenya" OR "Kiribati" OR "Kyrgyzstan" OR "Laos" OR "Lesotho" OR "Liberia" OR "Madagascar" OR "Malawi" OR "Mali" OR "Marshall Islands" OR "Mauritania" OR "Micronesia" OR "Mongolia" OR "Morocco" OR "Mozambique" OR "Myanmar" OR "Namibia" OR "Nauru" OR "Nepal" OR "Niger" OR "Nigeria" OR "Pakistan" OR "Papua New Guinea" OR "Philippines" OR "Rwanda" OR "Sao Tome and Principe" OR "Senegal" OR "Sierra Leone" OR "Solomon Islands" OR "Somalia" OR "South Africa" OR "South Sudan" OR "Sudan" OR "Swaziland" OR "Tajikistan" OR "Timor-Leste" OR "Togo" OR "Turkmenistan" OR "Uganda" OR "Tanzania" OR "Uzbekistan" OR "Yemen" OR "Zambia" OR "Zimbabwe" OR "developing country" OR "developing countries" OR "developing nation" OR "developing nations" OR "developing population" OR "developing populations" OR "developing world" OR "less developed country" OR "less developed countries" OR "less developed nation" OR "less developed nations" OR "less developed population" OR "less developed populations" OR "less developed world" OR "lesser developed country" OR "lesser developed countries" OR "lesser developed nation" OR "lesser developed nations" OR "lesser developed population" OR "lesser developed populations" OR "lesser developed world" OR "under developed country" OR "under developed countries" OR "under developed nation" OR "under developed nations" OR "under developed population" OR "under developed

populations" OR "under developed world" OR "underdeveloped country" OR "underdeveloped countries" OR "underdeveloped nation" OR "underdeveloped nations" OR "underdeveloped population" OR "underdeveloped populations" OR "underdeveloped world" OR "middle income country" OR "middle income countries" OR "middle income nation" OR "middle income nations" OR "middle income population" OR "middle income populations" OR "low income country" OR "low income countries" OR "low income nation" OR "low income nations" OR "low income population" OR "low income populations" OR "lower income country" OR "lower income countries" OR "lower income nation" OR "lower income nations" OR "lower income population" OR "lower income populations" OR "underserved country" OR "underserved countries" OR "underserved nation" OR "underserved nations" OR "underserved population" OR "underserved populations" OR "underserved world" OR "under served country" OR "under served countries" OR "under served nation" OR "under served nations" OR "under served population" OR "under served populations" OR "under served world" OR "deprived country" OR "deprived countries" OR "deprived nation" OR "deprived nations" OR "deprived population" OR "deprived populations" OR "deprived world" OR "poor country" OR "poor countries" OR "poor nation" OR "poor nations" OR "poor population" OR "poor populations" OR "poor world" OR "poorer country" OR "poorer countries" OR "poorer nation" OR "poorer nations" OR "poorer population" OR "poorer populations" OR "poorer world" OR "developing economy" OR "developing economies" OR "less developed economy" OR "less developed economies" OR "lesser developed economy" OR "lesser developed economies" OR "under developed economy" OR "under developed economies" OR "underdeveloped economy" OR "underdeveloped economies" OR "middle income economy" OR "middle income economies" OR "low income economy" OR "low income economies" OR "lower income economy" OR "lower income economies" OR "low gdp" OR "low gnp" OR "low gross domestic" OR "low gross national" OR "lower gdp" OR "lower gnp" OR "lower gross domestic" OR "lower gross national" OR "lmic" OR "lmics" OR "third world" OR "lami country" OR "lami countries" OR "transitional country" OR "transitional countries" OR "Africa" OR "Africa South of the Sahara" OR "Africa, Central" OR "Africa, Eastern" OR "Africa, Southern" OR "Africa, Western" OR "Africa, Northern" OR "Caribbean Region" OR "West Indies" OR "Central America" OR "Latin America" OR "South America" OR "Asia, Central" OR "Asia, Northern" OR "Asia, southeastern" OR "Asia, western" OR "middle east" OR "Asia" OR "far east" OR "Transcaucasia" OR "USSR" OR "Atlantic Islands" OR "Indian Ocean Islands" OR "Pacific Islands" OR "Micronesia" OR "Melanesia" OR "province" OR "provinces" OR "district" OR "districts" OR "prefecture" OR "prefectures" OR "county" OR "counties" OR "municipality" OR "municipalities")

Exclusion criteria for systematic review

The following criteria were used to screen articles captured in the systematic review. Articles were excluded if they fulfilled any of the following:

1. Did not disaggregated data between 5-19-year-olds.
2. Aggregated data beyond 24-year-olds.
3. Did not present cause of death data.
4. Included less than two specific causes of death.
5. Was not conducted in a high-mortality country without high-quality vital registration data.
6. Was not conducted using standardized verbal autopsy methods.
7. Had more than 25% of deaths with undefined causes.
8. Was not conducted in a general population.
9. Completed surveillance before 1980.
10. Did not have a reference period that was 12 months (±1 month) or did not have a study duration of more than 24 months.
11. Did not present primary data.

Collapsing into study input data points

In both LMM and HMM, datapoints were collapsed hierarchically by year, sex, and/or age, to contain at least 15 deaths and reduce idiosyncratic errors, and retained if they included at least two causes. To limit the influence of extremely large but less informative data points, in the HMM studies were dropped if they included more than 5,000 total deaths or had 25% or more deaths categorized as "undetermined". As a result, 12 data points were dropped among 5-9 (4·9%), 9 among 10-14 (3·6%), and 8 among 15-19 (2·9%). Moreover, data points with more than 1,000 deaths in which one cause represented more than 50% of total deaths were also dropped, but this only applied to 4 datapoints for 15-19 males with large fractions of interpersonal violence.

VR data used in the HMM

To enhance model stability, we included 30 high-quality VR data points in the HMM input. In particular, we identified countries with high-quality VR data in which the all-cause mortality rate between ages 5 and 20 (${}_{15}q_5$) was above 10 per 1,000 population (threshold between LMM and HMM countries) at some point between 2000-2019.[2] Next, for each of these countries, we selected the last year with empirical high-quality VR data in which ${}_{15}q_5 \geq 10$. The table below summarizes the high-quality VR data points included in the HMM input.

Table S3.2. Vital registration data used in high-mortality model

ISO3	Country name	Year	${}_{15}q_5$	Deaths 5-19	Age-sex group
COL	Colombia	2003	10·36	8,582	All except 15-19 males
ECU	Ecuador	2003	10·04	2,850	
GUY	Guyana	2014	11·28	185	
NIC	Nicaragua	2006	10·08	1,295	
PER	Peru	2002	10·01	5,933	All age-sex groups
PRY	Paraguay	2004	10·04	1,335	
SLV	El Salvador	2015	11·47	1,518	
VEN	Venezuela	2014	13·58	7,509	All except 15-19 males

Webappendix 3.3 Input data map

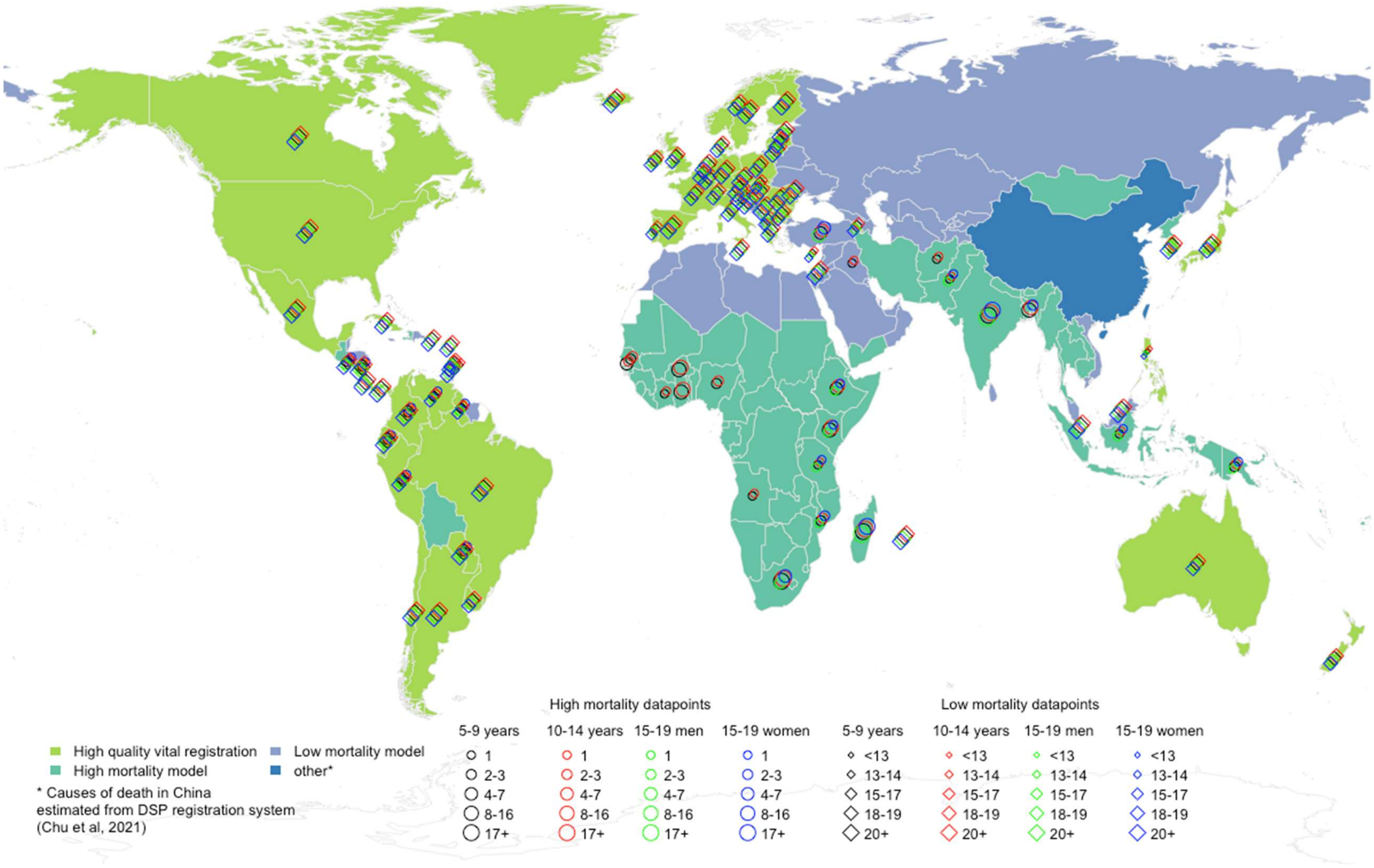

Note: The Chinese DSP comprises 5,648 site-years for ages 5-19, not shown in the map.

Webappendix 4. Additional details of modeling and estimation

Webappendix 4.1 The Bayesian LASSO

A thorough description of the Bayesian LASSO model used here can be found elsewhere.[9] A brief overview is provided in the following.

Suppose there exist C mutually exclusive causes of death, and that we have a sample of N_S deaths from a given study s, each of which is (correctly) classified into one and only one of the C categories. If we denote the distribution of true COD in the sample as $T_{1,s}, T_{2,s}, \ldots, T_{C,s}$ and if the sample is random, we can assume that these observations come from a multinomial distribution,

$$\begin{bmatrix} T_{1,s} \\ T_{2,s} \\ \vdots \\ T_{C,s} \end{bmatrix} \sim \text{Multinomial}\left(N_S, \begin{bmatrix} P_{1,s} \\ P_{2,s} \\ \vdots \\ P_{C,s} \end{bmatrix} \right),$$

where $P_{c,s}$ refers to the probability that a death is due to cause c in the population in which study s is conducted.

Suppose the probabilities $P_{c,s}$ can be predicted by the values of a set of K explanatory variables $X_{1,s}, X_{2,s}, \ldots, X_{K,s}$. In a multinomial regression framework, we assume that the logarithm of the odds of each cause of death relative to a reference cause are linearly dependent on these explanatory variables. This is expressed as a system of $C - 1$ linear equations corresponding to each cause of death (excluding the reference category $P_{1,s}$),

$$\begin{aligned} \log(P_{2,s}/P_{1,s}) &= \beta_{2,0} + \beta_{2,1}X_{1,s} + \beta_{2,2}X_{2,s} + \cdots + \beta_{2,K}X_{K,s} \\ \log(P_{3,s}/P_{1,s}) &= \beta_{3,0} + \beta_{3,1}X_{1,s} + \beta_{3,2}X_{2,s} + \cdots + \beta_{3,K}X_{K,s} \\ &\vdots \\ \log(P_{C,s}/P_{1,s}) &= \beta_{C,0} + \beta_{C,1}X_{1,s} + \beta_{C,2}X_{2,s} + \cdots + \beta_{C,K}X_{K,s} \end{aligned}$$

Note that the β-coefficients (including the intercepts) do not have the study subindex s. This is a fixed-effects model that assumes the associations of the explanatory variables with the causes of death are constant across all studies. We relax this assumption by adding study-specific normally distributed random effects to the intercepts, with mean 0 and cause-specific standard deviations. These standard deviations have uniformly distributed priors bounded between 0 and parameter b.

We implemented LASSO covariate selection by penalizing large β-coefficients in a subset of the fixed-effect parameters that could potentially result in overfitting the data. We did this by imposing a double exponential (also referred to as Laplace) prior distribution on them in the model specification.[10] This shrinks the magnitude of the parameters without completely reducing them to zero, and has the additional advantage of stabilizing the model if convergence is slow or difficult. The intercepts and any β-coefficient we did not want to be constrained in the LASSO were given non-informative normally distributed priors. The remaining β-coefficients had a Laplace prior with mean 0 and precision $\lambda > 0$, the penalty imposed by the LASSO method. We used out-of-sample cross-validation to select the optimal λ and b parameters (see Webappendix 9).

Once the model has estimated the β-coefficients and study-specific random effects, we estimated the expected distribution of true causes of death in any country for which we have covariate data as

$$P_{c,s} = \frac{\exp[U_{c,s} + \boldsymbol{\beta_c} \times \boldsymbol{X_s}]}{1 + \exp[U_{2,s} + \boldsymbol{\beta_2} \times \boldsymbol{X_s}] + \cdots + \exp[U_{C,s} + \beta_C \times \boldsymbol{X_s}]},$$

where $U_{c,s}$ denotes the cause- and study-specific random effect, $\boldsymbol{\beta_c}$ is a vector with β-coefficients for cause c, and $\boldsymbol{X_s}$ is the vector of covariates in study s.

The base category

For each age-sex group and model, selection of the base cause was guided by its global burden and input data availability. Specifically, we selected the base category by identifying the cause with the largest number of deaths in the input database.

Table S4.1. Base category for each age-sex group and model in the Bayesian LASSO

Model	Age-sex group	Base category
Low mortality model (LMM)	5-9 years	Neoplasms
	10-14 years	
	15-19 females	Road traffic injuries
	15-19 males	
High mortality model (HMM)	5-9 years	Diarrheal
	10-14 years	Lower respiratory infections
	15-19 females	Self-harm
	15-19 males	Road traffic injuries

Webappendix 4.2 Estimation of the sex-specific deaths and rates for 15-19

Country-level estimates on all-cause mortality (envelopes) were borrowed from the United Nations Inter-agency Group for Child Mortality Estimation (UN-IGME).[2] For the period 1990-2019 and ages 5 to 24, UN-IGME provides annual estimates on the number of deaths and mortality rates for 195 countries in 5- and 10-year age groups.[11]

However, UN-IGME has yet to publish sex-specific mortality envelopes for ages 5+. We used sex-specific life tables from the United Nations (UN)[12] to obtain all-cause sex-specific mortality rates and number of deaths for the 15-19 age group as follows:

1. For each country-year available, we recovered from the UN life tables the central death rates from 15 to 19 years for both sexes together (${}_5m_{15}$), for males (${}_5m_{15}^M$) and females (${}_5m_{15}^F$). Using these rates, for all country-years we calculated a male ratio $R^M = {}_5m_{15}^M / {}_5m_{15}$ and a female ratio $R^F = {}_5m_{15}^F / {}_5m_{15}$.
2. We transformed the UN-IGME probabilities of dying between ages 15 and 19 (${}_5q_{15}$) into central death rates by applying the formula ${}_5m_{15} = {}_5q_{15}/(5 \times (1000 - {}_5q_{15} \times 0.5))$, which assumes that deaths are equally distributed within each of the corresponding age intervals. Note

that UN-IGME reports probabilities of dying, even though they refer to them as ‘mortality rates’,[2] which justifies this intermediate step.

3. Next, we applied the ratios R^M and R^F calculated from the UN life tables to the estimated central death rates from UN-IGME (${}_5m_{15}$), obtaining the sex-specific death rates ${}_5m_{15}^M = R^M \times {}_5m_{15}$ and ${}_5m_{15}^F = R^F \times {}_5m_{15}$.
4. Finally, we back transformed these rates into sex-specific probabilities of dying, given by ${}_5q_{15}^M = 5000\ \times {}_5m_{15}^M/(1 + 2.5 \times {}_5m_{15}^M)$ and ${}_5q_{15}^F = 5000\ \times {}_5m_{15}^F/(1 + 2.5 \times {}_5m_{15}^F)$.

Similarly, we used the population shares by age and sex from UN World Population Prospects (UN-WPP)[12] to calculate the sex-specific number of deaths in the 15-19 age group.

5. For each country-year, we divided the reported number of deaths (${}_5D_{15}$) from UN-IGME by the estimated death rates ${}_5m_{15}$ defined above, obtaining the population at risk for both sexes ${}_5P_{15} = {}_5D_{15}/\ {}_5m_{15}$.
6. Let $Prop^M$ and $Prop^F$ denote the country- and year-specific shares by sex from UN-WPP for the 15-19 age group. We calculated the sex-specific population at risk for each country-year as ${}_5P_{15}^M = Prop^M \times {}_5P_{15}$ and ${}_5P_{15}^F = Prop^F \times {}_5P_{15}$.
7. Finally, we estimated the sex-specific number of deaths for the 15-19 age group multiplying the sex-specific populations at risk by the sex-specific death rates from UN-IGME estimated above: ${}_5D_1^M\ = {}_5P_{15}^M \times {}_5m_{15}^M$ and ${}_5D_{15}^F = {}_5P_{15}^F \times {}_5m_{15}^F$.

Webappendix 4.3 Point estimates and uncertainty

We run 10,000 iterations of the eight Bayesian models (LMM and HMM in each of the four age-sex groups) in four parallel chains. To assess convergence, we used an initial burn-in sequence of 4,000 and a thinning interval of 20 and calculated potential scale reduction factor.[13] Hence, for each of the model parameters—and for each of the eight models—we got 1,204 sets of estimates after burn-in. We used these sets to calculate mean values of the multinomial regression parameters and obtain point estimates of the cause-specific mortality fractions, mortality rates and death counts. To estimate uncertainty, we used the 1,204 sets of multinomial regression parameters from the Bayesian model to obtain 1,204 sets of mortality estimates for each country-year and calculate uncertainty intervals. More specifically, for each draw we

1. Calculated mortality fractions for all countries in the period 2000-2019;
2. Randomly drew all-cause mortality estimates from the posterior distribution of the mortality envelopes;[2]
3. Incorporated single-cause estimates. For each country-year, values of the single-cause estimates were drawn from the corresponding uncertainty intervals, with the following assumptions:
    a. For HIV/AIDS, we assumed data were normally distributed but truncated at 0, to avoid negative values. We used the point estimates as the means an estimated the standard deviations by dividing the range of the corresponding 95% uncertainty intervals by 3.92.
    b. For TB and measles, the upper bounds of the corresponding 95% uncertainty intervals tended to be large, indicating skewed distributions with long upper tails. To account for that, we drew values from log-normal distributions with matching means and standard deviations. Let a and b denote the lower and upper bounds of the 95% uncertainty intervals, respectively, and m the point estimate. We defined $s = (b - a)/3.92$, and used standard formulae to obtain the usual log-normal parameters mean μ and variance σ^2, given by

$$\mu = \log\left(\frac{m^2}{\sqrt{s^2 + m^2}}\right) \quad \text{and} \quad \sigma^2 = \log\left(1 + \frac{s^2}{m^2}\right) \; .$$

Webappendix 4.4 Transparency and replicability

We carried out our analyses using the open-source statistical software R[14] and Bayesian modelling was implemented in JAGS[15] with wrapper functions from the `R2jags` package.[16] The source code, primary inputs and cause of death data collected and estimated are publicly available for research purposes from the GitHub repository https://github.com/panchoVG/Mort5to19.

Webappendix 5. Modeling and estimation strategies

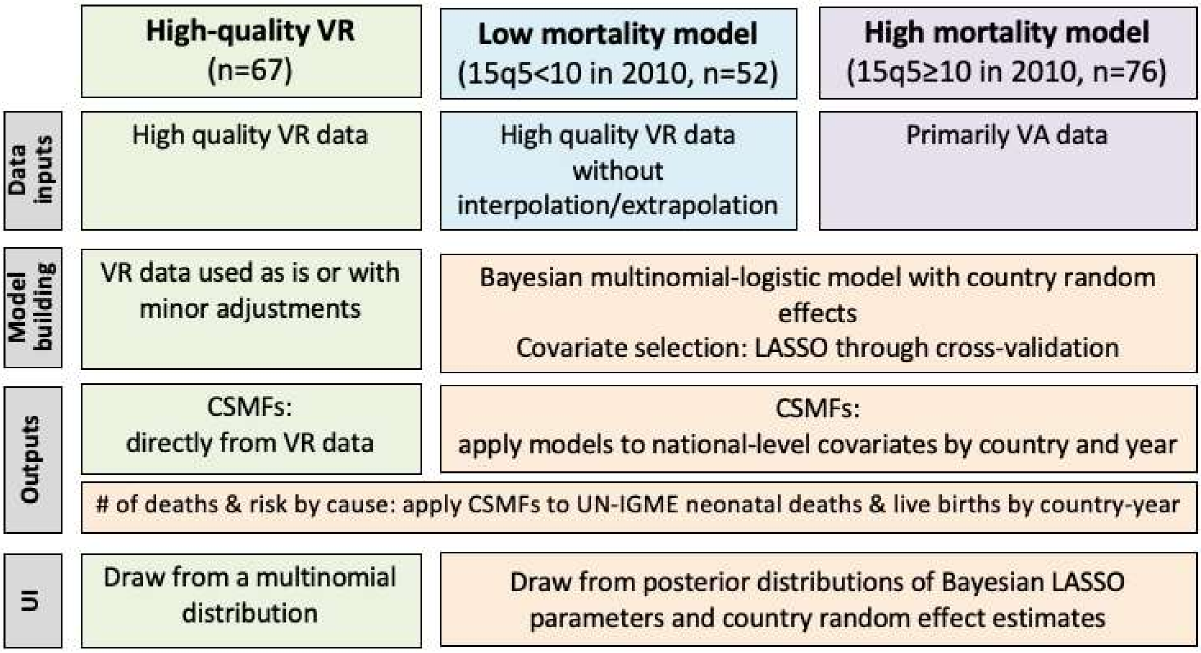

VR: Vital Registration; VA: Verbal Autopsy; LASSO: Least Absolute Shrinkage and Selection Operator; CSMF: Cause-Specific Mortality Fraction; UN-IGME: United Nations Inter-agency Group for Child Mortality Estimation

Webappendix 6. Additional details on single cause estimates

Webappendix 6.1 Splitting measles into endemic and epidemic

Measles estimates were taken from WHO Immunization, Vaccines and Biologicals Department.[17] These estimates assume zero deaths for ages 10 years and older. For 5-9 year-olds, we split measles deaths into endemic and epidemic deaths in countries where the measles caused at least 5% of total deaths in any year between 2000-2019 or more in this age group. Endemic measles was identified by fitting either a log-linear or loess model to the number of measles deaths and were accounted for within the UN-IGME all-cause mortality estimates, whereas epidemic measles was the difference between the total and endemic measles deaths, and were added outside the UN-IGME all-cause mortality estimates. Then, they were recombined to get the final measles cause of death fractions presented here.

The following countries had measles deaths that were calculated outside the UN envelope: Afghanistan, Angola, Benin, Burkina Faso, Bangladesh, Bhutan, Botswana, Central African Republic, Côte d'Ivoire, Cameroon, Democratic Republic of the Congo, Congo, Cabo Verde, Djibouti, Algeria, Egypt, Eritrea, Ethiopia, Fiji, Gabon, Ghana, Guinea, Gambia, Guinea-Bissau, Equatorial Guinea, Indonesia, India, Iraq, Cambodia, Lao People's Democratic Republic, Lebanon, Liberia, Madagascar, Mali, Myanmar, Mozambique, Mauritania, Niger, Nigeria, Nepal, Pakistan, Papua New Guinea, Sudan, Senegal, Sierra Leone, Somalia, South Sudan, Sao Tome and Principe, Chad, Togo, Uganda, Viet Nam, Samoa, Yemen, and Zambia.

Webappendix 6.2 The process of squeezing extrapulmonary TB, HIV/AIDS and endemic measles into Other CMPN

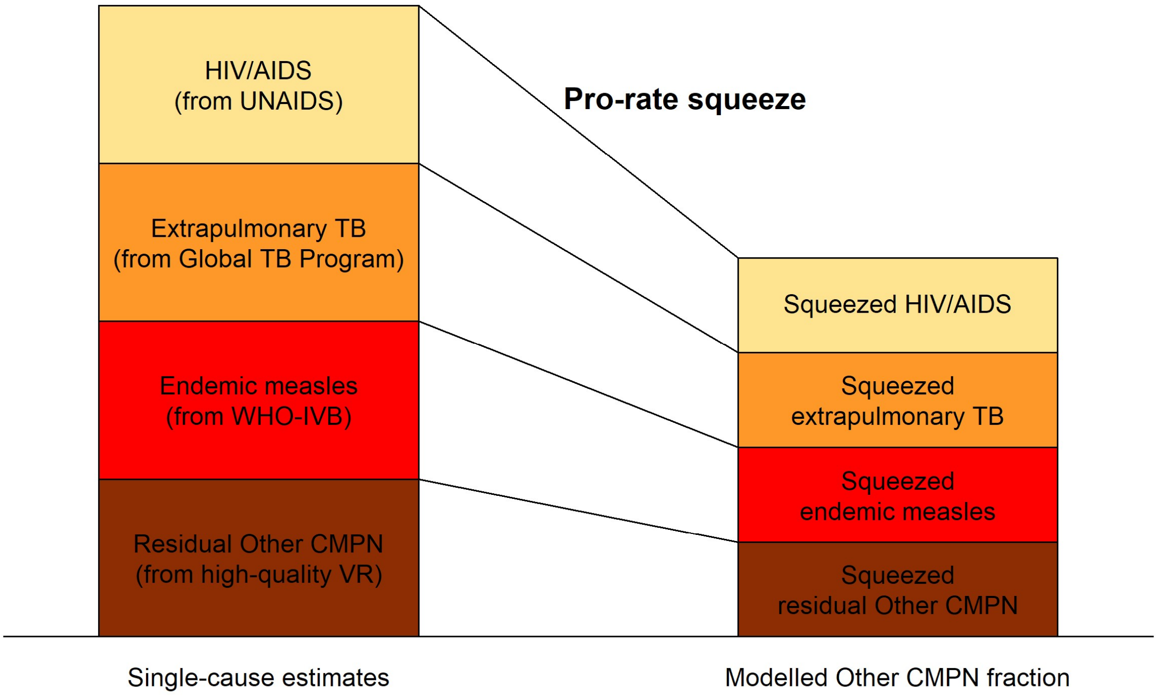

Webappendix 6.3 Crisis estimates

Crisis estimates, including collective violence, natural disasters, and infectious disease epidemics, were separately produced, and redistributed into causes of death categories depending on the nature of the crisis according to the table below. For a small number of country-years ($n = 750$) in which collective violence or natural disasters were implausibly high, we capped those cause fractions to the GHE fractions.[4]

Table S6.3. Cause categorization for crisis estimates

Cause	Type of crisis
Collective Violence	Civil war, war, conflict, genocide, invasion, protests, crisis, uprising, insurgency, war spillover, offensive
Natural Disasters	Earthquake, cyclone, flood and mudslides, hurricane and landslides, tsunami, forest fires
All causes	Unclear (Jordan 2005, Turkey 2016)
Other CMPN/measles/HIV/malaria/TB	Ebola/Measles

Webappendix 7. The GATHER checklist

Item #	Checklist item	Reported on section
**Objectives and funding**		
**1**	Define the indicator(s), populations (including age, sex, and geographic entities), and time period(s) for which estimates were made.	Introduction
**2**	List the funding sources for the work.	Abstract
**Data Inputs**		
For all data inputs from multiple sources that are synthesized as part of the study:		
**3**	Describe how the data were identified and how the data were accessed.	Methods
**4**	Specify the inclusion and exclusion criteria. Identify all ad-hoc exclusions.	Methods; Webappendix 3
**5**	Provide information on all included data sources and their main characteristics. For each data source used, report reference information or contact name/institution, population represented, data collection method, year(s) of data collection, sex and age range, diagnostic criteria or measurement method, and sample size, as relevant.	Methods; Webappendix 3
**6**	Identify and describe any categories of input data that have potentially important biases (e.g., based on characteristics listed in item 5).	Methods; Webappendix 3
For data inputs that contribute to the analysis but were not synthesized as part of the study:		
**7**	Describe and give sources for any other data inputs.	Methods; Webappendices 3 and 6
For all data inputs:		
**8**	Provide all data inputs in a file format from which data can be efficiently extracted (e.g., a spreadsheet rather than a PDF), including all relevant meta-data listed in item 5. For any data inputs that cannot be shared because of ethical or legal reasons, such as third-party ownership, provide a contact name or the name of the institution that retains the right to the data.	Primary inputs and cause of death data collected are publicly available from the GitHub repository https://github.com/panchoVG/Mort5to19.
**Data analysis**		
**9**	Provide a conceptual overview of the data analysis method. A diagram may be helpful.	Methods; Webappendix 5
**10**	Provide a detailed description of all steps of the analysis, including mathematical formulae. This description should cover, as relevant, data cleaning, data pre-processing, data adjustments and weighting of data sources, and mathematical or statistical model(s).	Methods; Webappendices 4 and 6
**11**	Describe how candidate models were evaluated and how the final model(s) were selected.	Methods; Webappendix 9
**12**	Provide the results of an evaluation of model performance, if done, as well as the results of any relevant sensitivity analysis.	Webappendix 9
**13**	Describe methods for calculating uncertainty of the estimates. State which sources of uncertainty were, and were not, accounted for in the uncertainty analysis.	Methods; Webappendix 4

Webappendix 8. Inputs and outputs by estimation methods

	Input Data				Output Estimates	
	Data points	Deaths	Covariates	Countries	Deaths 2019	Countries
**5-9-year-olds**						
High-quality VR data	2,570 (66·9%)	461,684 (47·5%)	-	67	19,980 (3·9%)	67
Low mortality model	1,041 (27·1%)	456,853 (47·0%)	11	58	22,928 (4·4%)	51
High mortality model	230 (6·0%)	54,013 (5·6%)	13	29	457,896 (88·6%)	76
China	5,648	36,366	-	1	16,167 (3·1%)	1
				Total	516,971	195
**10-14-year-olds**						
High-quality VR data	2,570 (66·6%)	538,941 (47·1%)	-	67	24,437 (6·6%)	67
Low mortality model	1,059 (27·4%)	533,766 (46·7%)	12	58	21,327 (5·8%)	51
High mortality model	230 (6·0%)	71,508 (6·3%)	13	29	306,276 (83·2%)	76
China	5,648	37,207	-	1	16,111 (4·4%)	1
				Total	368,151	195
**15-19-year-old females**						
High-quality VR data	1,285 (51·7%)	412,694 (48·8%)	-	67	19,773 (8·1%)	67
Low mortality model	1,050 (42·2%)	409,483 (48·4%)	13	57	12,768 (5·3%)	51
High mortality model	153 (6·2%)	23,637 (2·8%)	12	20	202,095 (83·1%)	76
China	5,648	43,650	-	1	8,589 (3·5%)	1
				Total	243,225	195
**15-19-year-old males**						
High-quality VR data	1,285 (51·0%)	1,143,840 (49·6%)	-	67	55,612 (15·8%)	67
Low mortality model	1,091 (43·3%)	1,136,458 (49·3%)	14	58	27,391 (7·8%)	51
High mortality model	145 (5·8%)	26,782 (1·2%)	11	17	255,152 (72·5%)	76
China	5,648	18,129			13,726 (3·9%)	1
				Total	351,881	195
**5-19-year-olds**						
High-quality VR data					119,802 (8·1%)	67
Low mortality model					84,414 (5·7%)	51
High mortality model					1,221,418 (82·5%)	76
China					54,593 (3·7%)	1
				Total	1,480,227	195

[a] Percentages calculated within each age-sex group. China is excluded from the percentage calculations for inputs since its data was not included in the modeling.
[b] Estimates for China are derived through adjusting empirical data, not modeling (Liu Y, Chu Y, Yeung D *et al.*; unpublished data).
[c] Inputs for LMM are high-quality VR country-years with at least 15 total deaths. Countries with less than 15 deaths for the whole period (2000-2019) were not represented in the input data.

Webappendix 9. Model selection process

Identification of LASSO precision parameter λ through cross-validation by estimation methods. Based on out-of-sample prediction with a random effect standard deviation set at $b = 0 \cdot 07$, the best λ, defined as the one that gives the least root mean squared error in the out-of-sample prediction, is marked red in the figures below.

Webappendix 9.1 Cross-validation for low-mortality model (LMM)

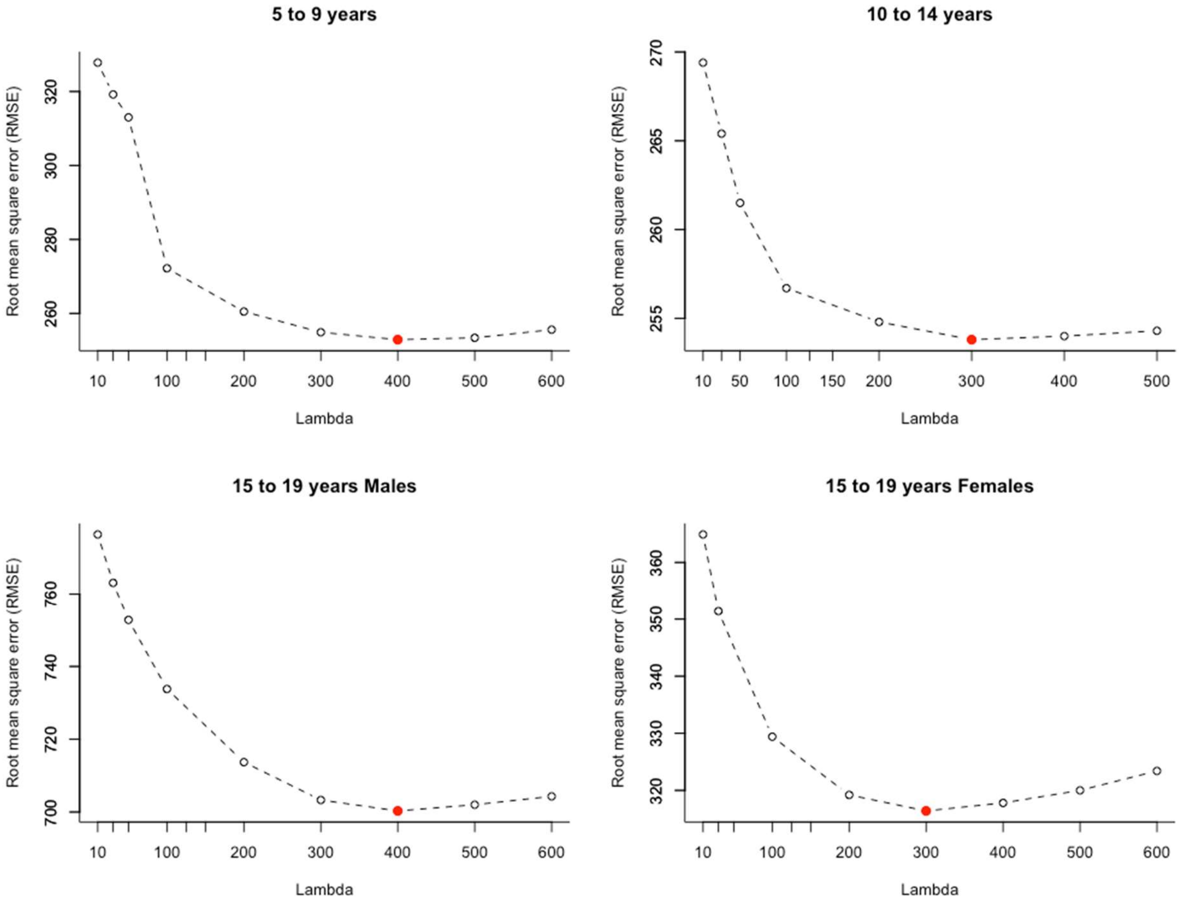

Webappendix 9.2 Cross-validation for high-mortality model (HMM)

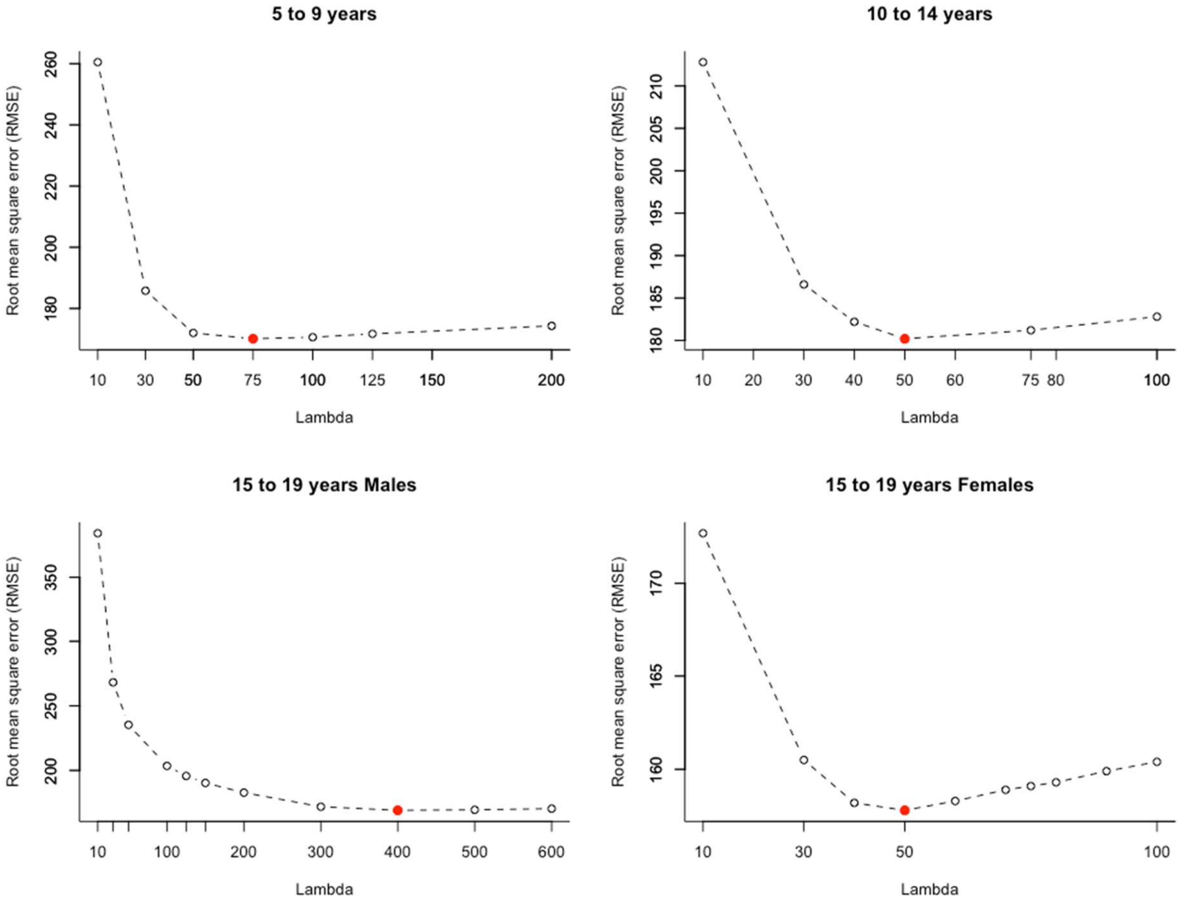

Webappendix 10. Global and regional cause-specific mortality estimates by age-sex group, 2000-2019

Webappendix 10.1 Data files with global and regional cause-specific mortality estimates

The following CSV (comma separated value) files with mortality estimates are available from the GitHub repository https://github.com/panchoVG/Mort5to19.

Mortality estimates without uncertainty (point estimates)

- 'PointEstimates5to9-Regional.csv': Global and regional all-cause number of deaths, all-cause mortality rates, and cause-specific mortality fractions for 5-9 years, 2000-2019.
- 'PointEstimates10to14-Regional.csv': Global and regional all-cause number of deaths, all-cause mortality rates, and cause-specific mortality fractions for 10-14 years, 2000-2019.
- 'PointEstimates15to19-Regional.csv': Global and regional all-cause number of deaths, all-cause mortality rates, and cause-specific mortality fractions for 15-19 years, 2000-2019.
- 'PointEstimates5to19-Regional.csv': Global and regional all-cause number of deaths, all-cause mortality rates, and cause-specific mortality fractions for 5-19 years, 2000-2019.

Mortality estimates with 95% uncertainty intervals

- 'AllCauseUncert-Regional.csv': Global and regional all-cause number of deaths and all-cause mortality rates with 95% uncertainty intervals, 5-9, 10-14 and 15-19 years, 2000-2019.
- 'Uncertainty5to9-Regional.csv': Global and regional cause-specific number of deaths, mortality rates, and mortality fractions with 95% uncertainty intervals for 5-9 years, 2000-2019.
- 'Uncertainty10to14-Regional.csv': Global and regional cause-specific number of deaths, mortality rates, and mortality fractions with 95% uncertainty intervals for 10-14 years, 2000-2019.
- 'Uncertainty15to19-Regional.csv': Global and regional cause-specific number of deaths, mortality rates, and mortality fractions with 95% uncertainty intervals for 15-19 years, 2000-2019.
- 'Uncertainty5to19-Regional.csv': Global and regional cause-specific number of deaths and mortality fractions with 95% uncertainty intervals for 5 to 19 years, 2000-2019.
- 'GlobalAARR.csv': Global cause-specific annual average rate of reduction (AARR) 2000-2019 with 95% uncertainty intervals, 5-9, 10-14 and 15-19 years.

Webappendix 10.2 Global and regional leading causes of death 5-19 years in 2019

Table S10.2. Global and regional leading causes of death 5-19 years in 2019

Region	Cause	Number of deaths	Fraction
World	Road traffic injuries	115,843 (110,672-125,054)	7·8% (7·5-8·1%)
West and central Africa	Malaria	63,205 (53,960-75,377)	14·4% (12·9-15·7%)
Eastern and Southern Africa	HIV/AIDS	19,883 (17,267-24,673)	6·6% (5·9-7·4%)
Middle East and North Africa	Collective violence	14,359 (11,442-18,436)	20·1% (17-23·3%)
South Asia	Road traffic injuries	30,081 (26,047-36,080)	8·2% (7·6-8·7%)
East Asia and Pacific	Neoplasms	19,972 (17,242-24,521)	12·4% (11·6-13·4%)
Latin America and Caribbean	Interpersonal violence	18,029 (17,155-19,021)	21·6% (20·6-22·4%)
North America	Road traffic injuries	3,741 (3,490-3,998)	20·8% (20·1-21·4%)
Eastern Europe and central Asia	Neoplasms	4,221 (4,112-4,334)	16·6% (16·3-16·9%)
Western Europe	Neoplasms	2,050 (1,968-2,147)	19·8% (19·1-20·5%)

Note: Values between parenthesis denote 95% uncertainty intervals.

Webappendix 10.3 Global and regional causes of mortality fractions by age-sex group in 2019

The following table contains the data for Figure 3 from the main manuscript.

Table S10.3. Global and regional causes of mortality fractions by age-sex group in 2019

Region	Cause of death	5 to 9	10 to 14	15 to 19 females	15 to 19 males
World	Maternal	NA	NA	5.00%	NA
	Measles	4.21%	NA	NA	NA
	HIV/AIDS	1.62%	3.28%	2.45%	2.03%
	LRI	6.55%	6.80%	NA	NA
	TB	4.33%	2.12%	6.32%	7.18%
	Diarrhoeal	9.99%	5.72%	NA	NA
	Malaria	9.66%	8.58%	NA	NA
	Other CMPN	16.82%	16.82%	11.70%	11.72%
	Congenital	3.40%	NA	NA	NA
	Cardiovascular	NA	NA	6.97%	6.16%
	Digestive system	3.12%	2.88%	5.32%	4.74%
	Neoplasms	5.50%	7.28%	7.52%	6.20%
	Other NCD	13.10%	15.86%	19.37%	12.15%
	Interpersonal violence	NA	NA	4.29%	12.25%
	Self-harm	NA	NA	13.42%	8.31%
	Drowning	6.40%	6.41%	1.86%	4.61%
	RTI	5.41%	6.12%	6.81%	13.86%
	Other injuries	8.86%	16.45%	7.55%	8.79%
	Natural disasters	0.06%	0.08%	0.05%	0.07%
	Collective violence	0.98%	1.58%	1.36%	1.93%
West and central Africa	Maternal	NA	NA	6.73%	NA
	Measles	5.07%	NA	NA	NA
	HIV/AIDS	1.49%	3.84%	2.87%	3.04%
	LRI	8.73%	9.46%	NA	NA
	TB	3.20%	1.50%	8.03%	12.24%
	Diarrhoeal	13.48%	11.36%	NA	NA
	Malaria	19.18%	22.17%	NA	NA
	Other CMPN	25.40%	29.39%	25.53%	20.93%
	Congenital	2.79%	NA	NA	NA
	Cardiovascular	NA	NA	8.08%	6.29%
	Digestive system	1.96%	1.70%	6.03%	5.11%
	Neoplasms	2.47%	2.34%	4.40%	4.81%
	Other NCD	5.42%	8.08%	13.14%	9.81%
	Interpersonal violence	NA	NA	2.70%	6.25%
	Self-harm	NA	NA	9.01%	6.91%
	Drowning	2.91%	2.94%	1.14%	3.90%
	RTI	4.36%	3.53%	5.18%	10.76%
	Other injuries	3.22%	3.21%	6.26%	8.19%
	Natural disasters	0.00%	0.00%	0.00%	0.00%
	Collective violence	0.30%	0.47%	0.89%	1.74%
Eastern and Southern Africa	Maternal	NA	NA	4.85%	NA
	Measles	3.67%	NA	NA	NA
	HIV/AIDS	3.75%	9.93%	8.43%	6.66%
	LRI	8.63%	11.47%	NA	NA
	TB	3.73%	1.87%	7.40%	7.83%
	Diarrhoeal	10.43%	6.10%	NA	NA
	Malaria	9.12%	9.06%	NA	NA

Region	Cause of death	5 to 9	10 to 14	15 to 19 females	15 to 19 males
	Other CMPN	17.94%	18.96%	14.44%	17.97%
	Congenital	2.82%	NA	NA	NA
	Cardiovascular	NA	NA	7.16%	6.53%
	Digestive system	3.18%	2.54%	6.20%	5.66%
	Neoplasms	3.75%	5.29%	5.62%	5.31%
	Other NCD	14.10%	14.03%	17.10%	10.89%
	Interpersonal violence	NA	NA	3.69%	5.49%
	Self-harm	NA	NA	11.23%	7.66%
	Drowning	5.51%	6.02%	1.41%	4.31%
	RTI	4.54%	4.60%	4.74%	11.90%
	Other injuries	8.55%	9.71%	7.15%	8.97%
	Natural disasters	0.10%	0.14%	0.08%	0.10%
	Collective violence	0.18%	0.28%	0.49%	0.72%
Middle East and North Africa	Maternal	NA	NA	3.77%	NA
	Measles	2.38%	NA	NA	NA
	HIV/AIDS	0.12%	0.10%	0.13%	0.07%
	LRI	5.78%	4.30%	NA	NA
	TB	1.19%	0.42%	1.49%	1.00%
	Diarrhoeal	3.06%	1.71%	NA	NA
	Malaria	0.24%	0.17%	NA	NA
	Other CMPN	8.78%	9.00%	6.72%	10.91%
	Congenital	6.02%	NA	NA	NA
	Cardiovascular	NA	NA	4.58%	4.67%
	Digestive system	2.19%	1.72%	3.13%	2.26%
	Neoplasms	13.47%	13.24%	9.33%	6.93%
	Other NCD	16.05%	20.22%	26.77%	12.87%
	Interpersonal violence	NA	NA	6.81%	16.79%
	Self-harm	NA	NA	11.22%	5.61%
	Drowning	5.31%	3.88%	0.95%	3.48%
	RTI	7.16%	5.72%	5.23%	11.86%
	Other injuries	10.72%	13.44%	2.99%	4.20%
	Natural disasters	0.03%	0.03%	0.02%	0.02%
	Collective violence	17.49%	26.06%	16.88%	19.34%
South Asia	Maternal	NA	NA	4.70%	NA
	Measles	4.85%	NA	NA	NA
	HIV/AIDS	0.21%	0.35%	0.04%	0.04%
	LRI	1.91%	2.98%	NA	NA
	TB	7.87%	3.48%	7.40%	9.55%
	Diarrhoeal	8.65%	3.04%	NA	NA
	Malaria	0.39%	0.42%	NA	NA
	Other CMPN	6.90%	9.43%	4.00%	8.14%
	Congenital	2.71%	NA	NA	NA
	Cardiovascular	NA	NA	6.19%	7.22%
	Digestive system	5.73%	4.70%	6.03%	7.18%
	Neoplasms	5.02%	6.93%	7.54%	5.90%
	Other NCD	20.20%	16.07%	21.45%	13.67%
	Interpersonal violence	NA	NA	4.27%	9.03%
	Self-harm	NA	NA	18.88%	8.65%
	Drowning	12.44%	9.56%	2.44%	4.98%
	RTI	5.54%	7.84%	5.94%	13.84%
	Other injuries	17.06%	34.74%	10.21%	10.76%
	Natural disasters	0.05%	0.05%	0.03%	0.04%
	Collective violence	0.46%	0.42%	0.88%	1.00%
East Asia and Pacific	Maternal	NA	NA	3.64%	NA
	Measles	3.40%	NA	NA	NA

Region	Cause of death	5 to 9	10 to 14	15 to 19 females	15 to 19 males
	HIV/AIDS	1.19%	0.82%	0.41%	0.50%
	LRI	3.54%	3.78%	NA	NA
	TB	5.64%	2.90%	3.95%	6.16%
	Diarrhoeal	4.15%	1.42%	NA	NA
	Malaria	0.38%	0.25%	NA	NA
	Other CMPN	8.07%	8.03%	4.54%	6.05%
	Congenital	5.40%	NA	NA	NA
	Cardiovascular	NA	NA	8.38%	7.41%
	Digestive system	2.69%	2.94%	3.23%	4.05%
	Neoplasms	12.93%	15.62%	13.16%	8.82%
	Other NCD	20.01%	26.61%	24.53%	14.57%
	Interpersonal violence	NA	NA	4.10%	10.54%
	Self-harm	NA	NA	13.62%	9.10%
	Drowning	11.01%	10.92%	3.49%	6.97%
	RTI	8.81%	8.88%	9.26%	15.74%
	Other injuries	12.51%	17.56%	7.51%	9.82%
	Natural disasters	0.19%	0.17%	0.11%	0.14%
	Collective violence	0.07%	0.09%	0.08%	0.12%
Latin America and Caribbean	Maternal	NA	NA	5.95%	NA
	Measles	0.00%	NA	NA	NA
	HIV/AIDS	0.63%	0.67%	1.12%	0.50%
	LRI	5.66%	4.33%	NA	NA
	TB	1.01%	0.46%	1.35%	0.55%
	Diarrhoeal	2.80%	0.99%	NA	NA
	Malaria	0.03%	0.04%	NA	NA
	Other CMPN	9.05%	7.10%	8.33%	4.78%
	Congenital	7.22%	NA	NA	NA
	Cardiovascular	NA	NA	5.97%	3.39%
	Digestive system	3.07%	2.90%	3.55%	1.49%
	Neoplasms	18.12%	15.68%	10.96%	5.69%
	Other NCD	24.35%	30.01%	22.76%	9.33%
	Interpersonal violence	NA	NA	11.16%	40.91%
	Self-harm	NA	NA	9.61%	6.33%
	Drowning	4.96%	5.13%	1.29%	3.59%
	RTI	9.58%	10.46%	12.72%	16.29%
	Other injuries	13.08%	21.79%	4.98%	7.02%
	Natural disasters	0.44%	0.43%	0.26%	0.13%
	Collective violence	0.00%	0.00%	0.00%	0.00%
North America	Maternal	NA	NA	0.98%	NA
	Measles	0.00%	NA	NA	NA
	HIV/AIDS	0.00%	0.02%	0.07%	0.02%
	LRI	2.25%	1.49%	NA	NA
	TB	0.01%	0.00%	0.04%	0.01%
	Diarrhoeal	0.31%	0.19%	NA	NA
	Malaria	0.00%	0.00%	NA	NA
	Other CMPN	4.18%	2.80%	2.62%	1.38%
	Congenital	8.50%	NA	NA	NA
	Cardiovascular	NA	NA	4.41%	3.16%
	Digestive system	1.52%	1.68%	1.12%	0.58%
	Neoplasms	19.58%	15.35%	8.38%	5.39%
	Other NCD	27.75%	33.20%	24.40%	16.62%
	Interpersonal violence	NA	NA	7.56%	19.10%
	Self-harm	NA	NA	18.05%	22.74%
	Drowning	5.30%	3.21%	0.73%	2.75%
	RTI	14.35%	13.81%	27.85%	22.60%

Region	Cause of death	5 to 9	10 to 14	15 to 19 females	15 to 19 males
	Other injuries	16.00%	28.15%	3.73%	5.59%
	Natural disasters	0.26%	0.10%	0.05%	0.05%
	Collective violence	0.00%	0.00%	0.00%	0.00%
Eastern Europe and central Asia	Maternal	NA	NA	1.85%	NA
	Measles	0.10%	NA	NA	NA
	HIV/AIDS	0.91%	0.38%	0.54%	0.20%
	LRI	5.90%	5.64%	NA	NA
	TB	2.33%	0.63%	1.69%	1.25%
	Diarrhoeal	1.41%	0.66%	NA	NA
	Malaria	0.00%	0.00%	NA	NA
	Other CMPN	6.96%	4.95%	5.89%	4.62%
	Congenital	8.93%	NA	NA	NA
	Cardiovascular	NA	NA	7.06%	5.35%
	Digestive system	2.49%	2.46%	2.22%	1.28%
	Neoplasms	21.31%	18.95%	16.28%	11.30%
	Other NCD	23.13%	33.55%	23.87%	15.10%
	Interpersonal violence	NA	NA	4.70%	6.78%
	Self-harm	NA	NA	11.69%	13.70%
	Drowning	6.27%	6.85%	1.90%	6.07%
	RTI	9.18%	8.68%	16.13%	24.28%
	Other injuries	10.71%	16.88%	5.83%	9.37%
	Natural disasters	0.01%	0.02%	0.02%	0.02%
	Collective violence	0.36%	0.33%	0.33%	0.69%
Western Europe	Maternal	NA	NA	0.18%	NA
	Measles	0.02%	NA	NA	NA
	HIV/AIDS	0.01%	0.01%	0.16%	0.04%
	LRI	2.90%	2.55%	NA	NA
	TB	0.03%	0.07%	0.02%	0.05%
	Diarrhoeal	0.34%	0.13%	NA	NA
	Malaria	0.02%	0.01%	NA	NA
	Other CMPN	3.49%	2.67%	4.50%	2.86%
	Congenital	11.12%	NA	NA	NA
	Cardiovascular	NA	NA	5.76%	4.67%
	Digestive system	1.47%	1.56%	1.37%	0.85%
	Neoplasms	30.80%	25.73%	17.65%	12.79%
	Other NCD	31.10%	40.77%	28.44%	20.78%
	Interpersonal violence	NA	NA	2.17%	2.42%
	Self-harm	NA	NA	16.14%	19.27%
	Drowning	2.60%	2.57%	0.94%	3.63%
	RTI	6.74%	8.71%	16.38%	23.23%
	Other injuries	9.11%	14.96%	6.17%	9.35%
	Natural disasters	0.21%	0.23%	0.12%	0.07%
	Collective violence	0.02%	0.00%	0.00%	0.00%

Webappendix 10.4 Regional cause-specific mortality rates by age-sex group, time trends 2000-2019

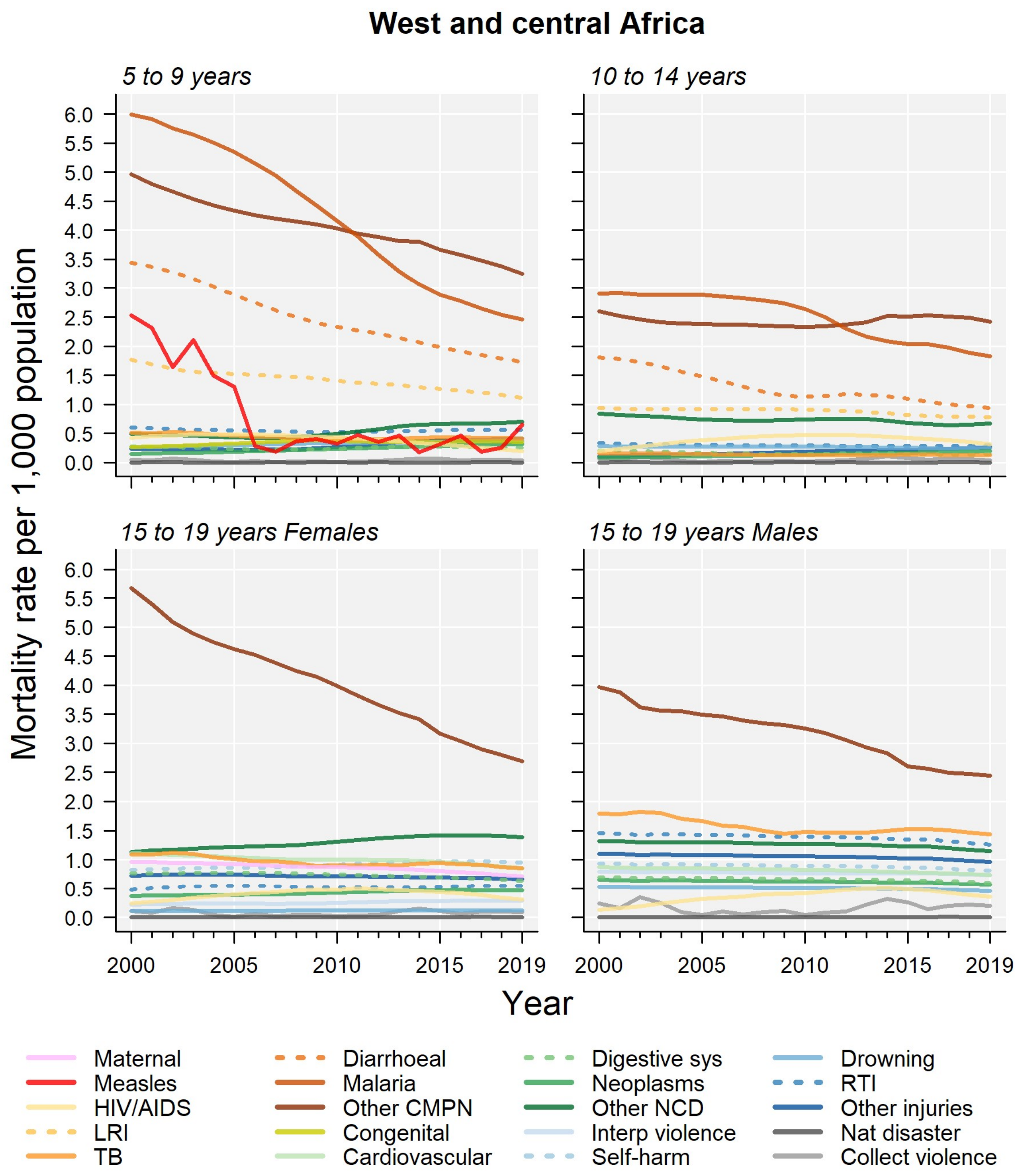

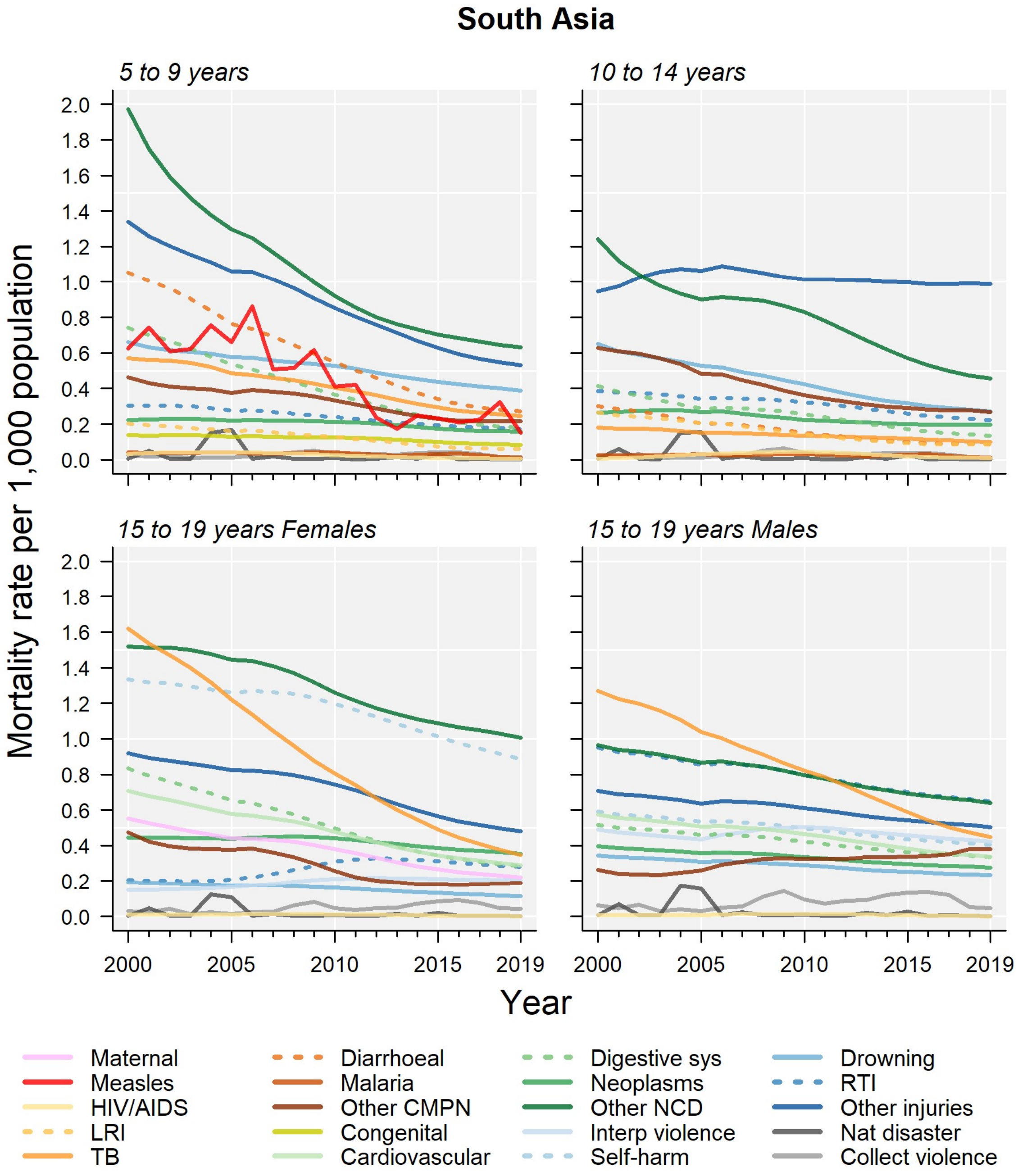
South Asia
5 to 9 years
10 to 14 years
15 to 19 years Females
15 to 19 years Males
Mortality rate per 1,000 population
0.0
0.2
0.4
0.6
0.8
1.0
1.2
1.4
1.6
1.8
2.0
2000
2005
2010
2015
2019
Year
Maternal
Measles
HIV/AIDS
LRI
TB
Diarrhoeal
Malaria
Other CMPN
Congenital
Cardiovascular
Digestive sys
Neoplasms
Other NCD
Interp violence
Self-harm
Drowning
RTI
Other injuries
Nat disaster
Collect violence

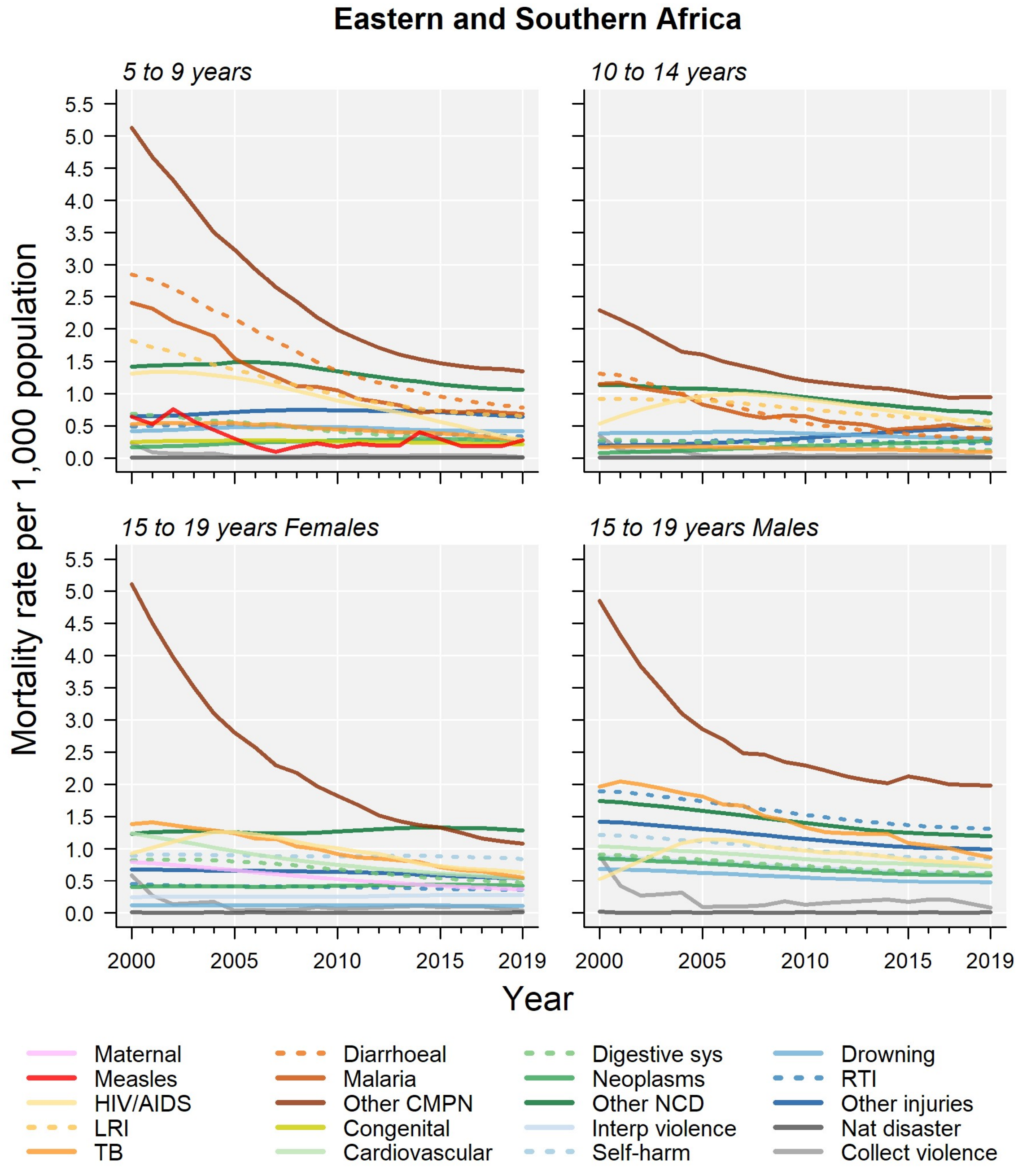
Eastern and Southern Africa
5 to 9 years
10 to 14 years
15 to 19 years Females
15 to 19 years Males
Mortality rate per 1,000 population
0.0
0.5
1.0
1.5
2.0
2.5
3.0
3.5
4.0
4.5
5.0
5.5
2000
2005
2010
2015
2019
Year
Maternal
Measles
HIV/AIDS
LRI
TB
Diarrhoeal
Malaria
Other CMPN
Congenital
Cardiovascular
Digestive sys
Neoplasms
Other NCD
Interp violence
Self-harm
Drowning
RTI
Other injuries
Nat disaster
Collect violence

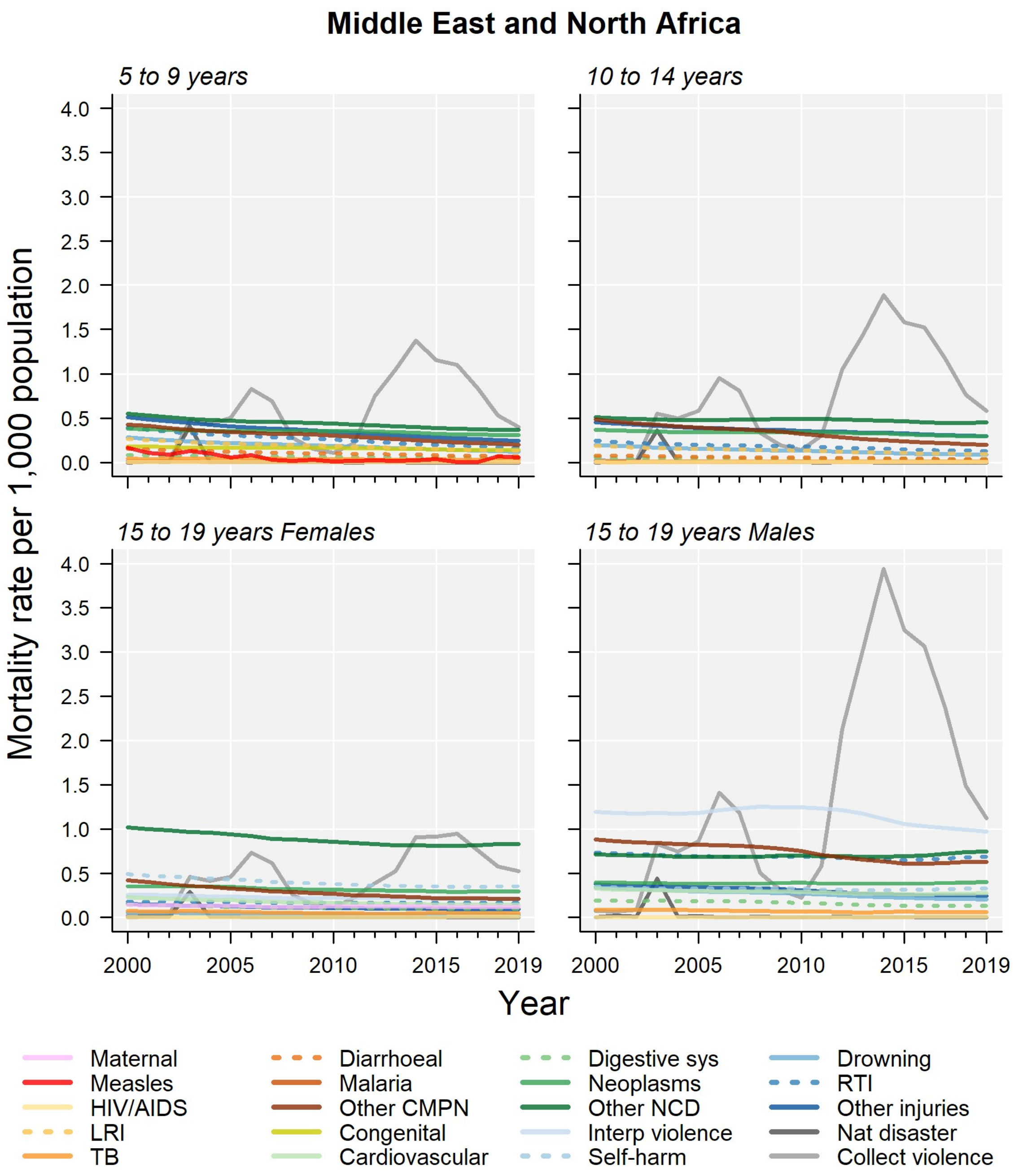
Middle East and North Africa
5 to 9 years
10 to 14 years
15 to 19 years Females
15 to 19 years Males
Mortality rate per 1,000 population
4.0
3.5
3.0
2.5
2.0
1.5
1.0
0.5
0.0
2000
2005
2010
2015
2019
Year
Maternal
Measles
HIV/AIDS
LRI
TB
Diarrhoeal
Malaria
Other CMPN
Congenital
Cardiovascular
Digestive sys
Neoplasms
Other NCD
Interp violence
Self-harm
Drowning
RTI
Other injuries
Nat disaster
Collect violence

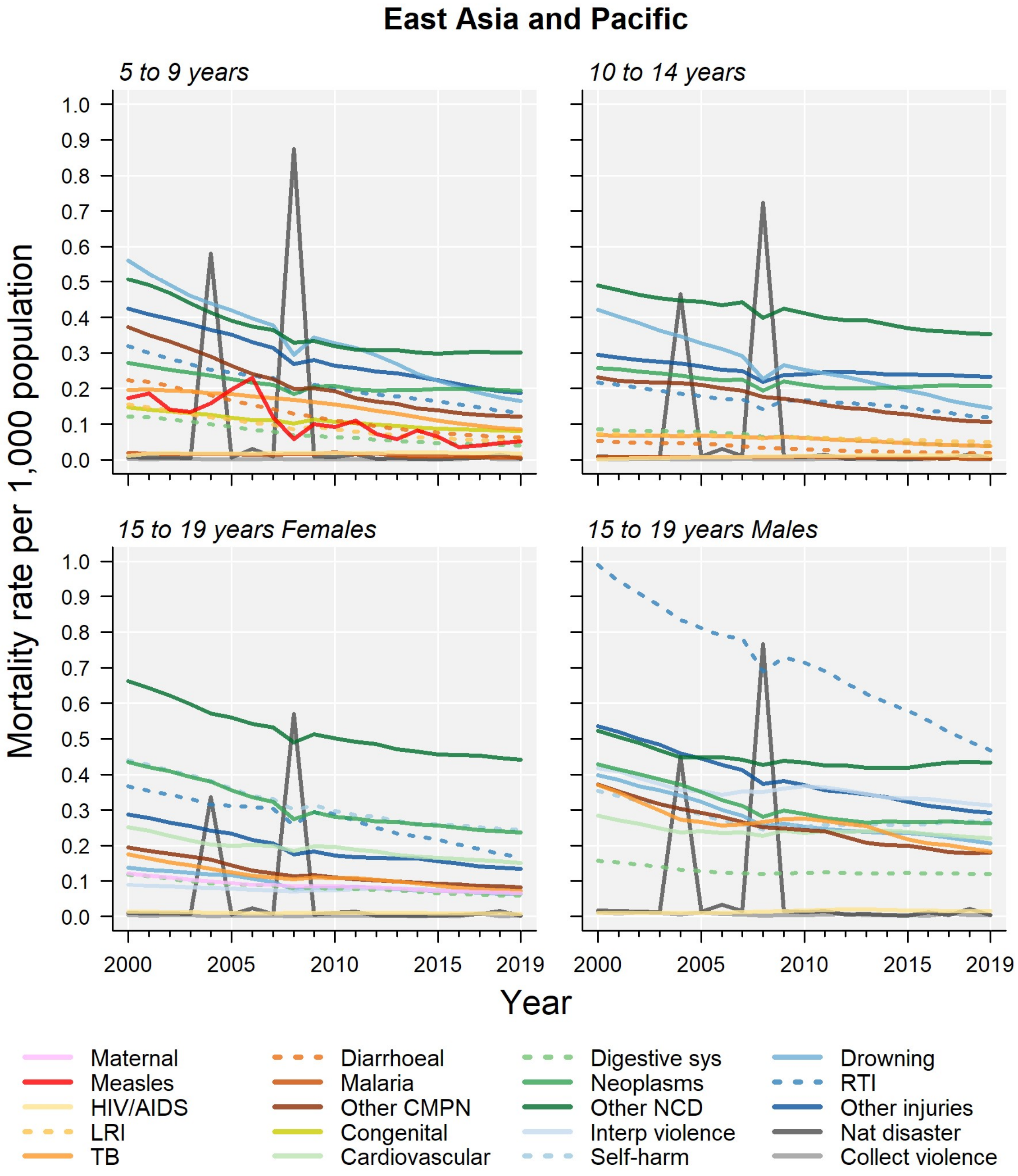
East Asia and Pacific
5 to 9 years
10 to 14 years
15 to 19 years Females
15 to 19 years Males
Mortality rate per 1,000 population
1.0
0.9
0.8
0.7
0.6
0.5
0.4
0.3
0.2
0.1
0.0
2000
2005
2010
2015
2019
Year
Maternal
Measles
HIV/AIDS
LRI
TB
Diarrhoeal
Malaria
Other CMPN
Congenital
Cardiovascular
Digestive sys
Neoplasms
Other NCD
Interp violence
Self-harm
Drowning
RTI
Other injuries
Nat disaster
Collect violence

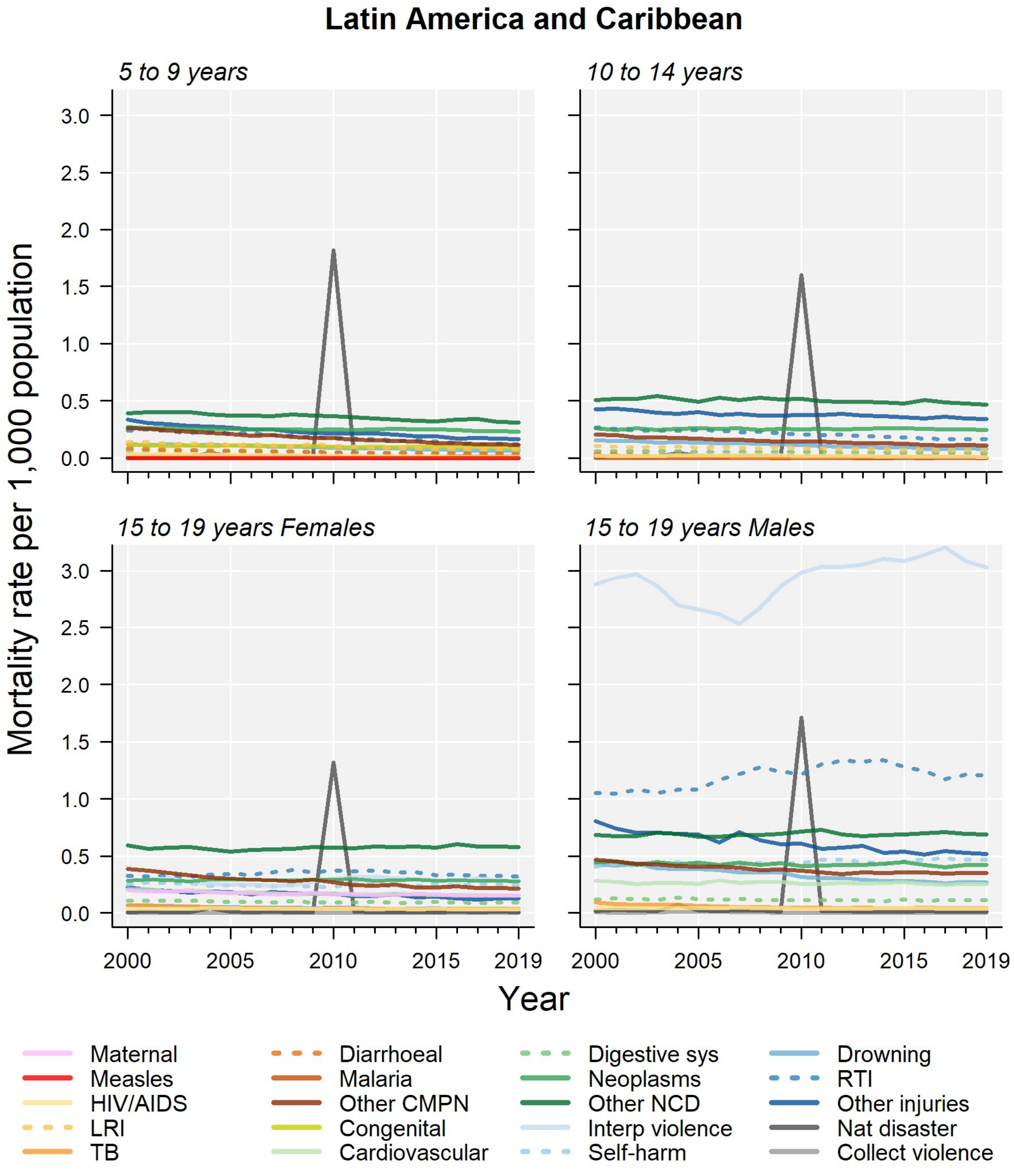
Latin America and Caribbean
5 to 9 years
10 to 14 years
15 to 19 years Females
15 to 19 years Males
Mortality rate per 1,000 population
0.0
0.5
1.0
1.5
2.0
2.5
3.0
2000
2005
2010
2015
2019
Year
Maternal
Measles
HIV/AIDS
LRI
TB
Diarrhoeal
Malaria
Other CMPN
Congenital
Cardiovascular
Digestive sys
Neoplasms
Other NCD
Interp violence
Self-harm
Drowning
RTI
Other injuries
Nat disaster
Collect violence

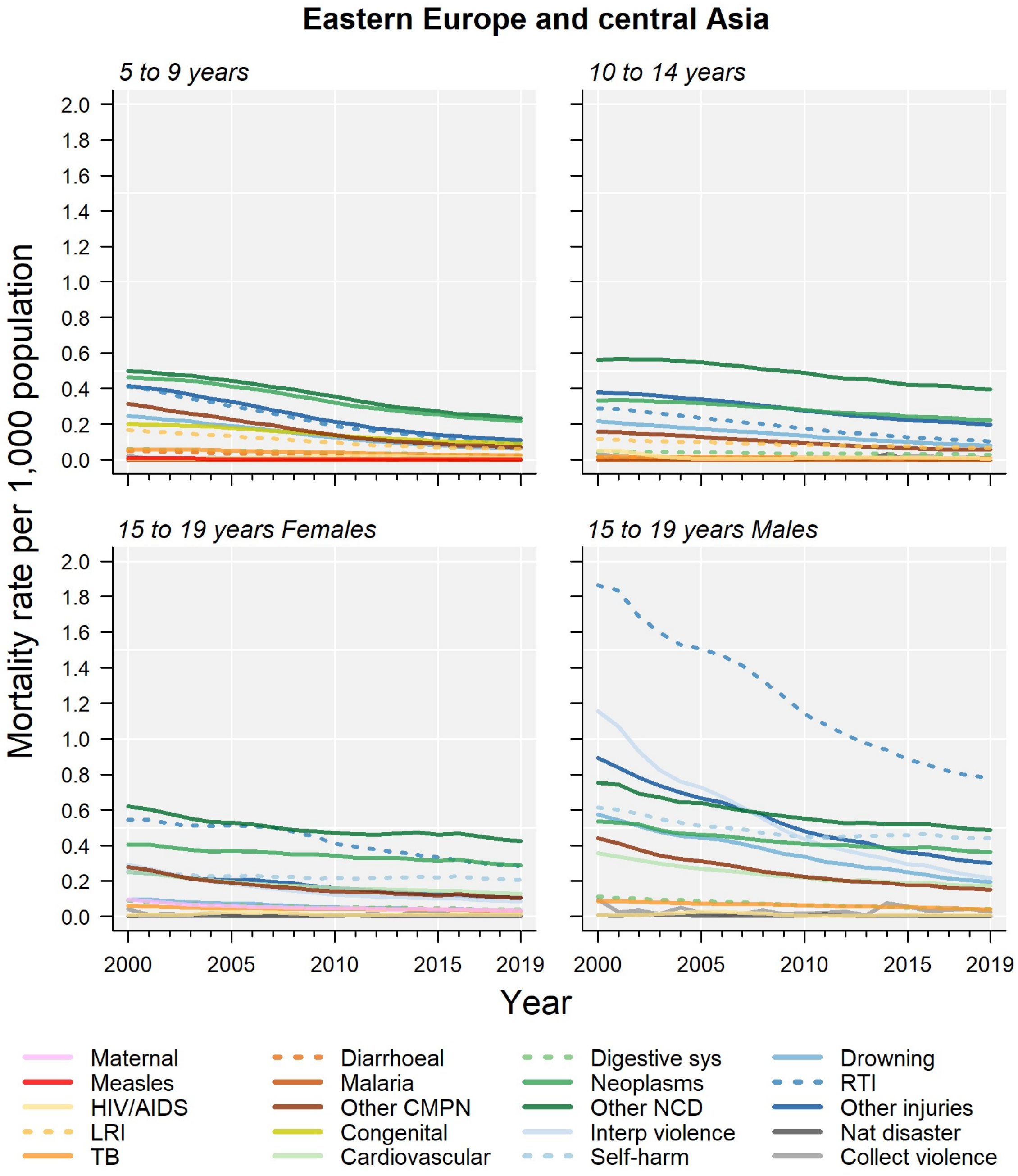
Eastern Europe and central Asia
5 to 9 years
10 to 14 years
15 to 19 years Females
15 to 19 years Males
Mortality rate per 1,000 population
2.0
1.8
1.6
1.4
1.2
1.0
0.8
0.6
0.4
0.2
0.0
2000
2005
2010
2015
2019
Year
Maternal
Measles
HIV/AIDS
LRI
TB
Diarrhoeal
Malaria
Other CMPN
Congenital
Cardiovascular
Digestive sys
Neoplasms
Other NCD
Interp violence
Self-harm
Drowning
RTI
Other injuries
Nat disaster
Collect violence

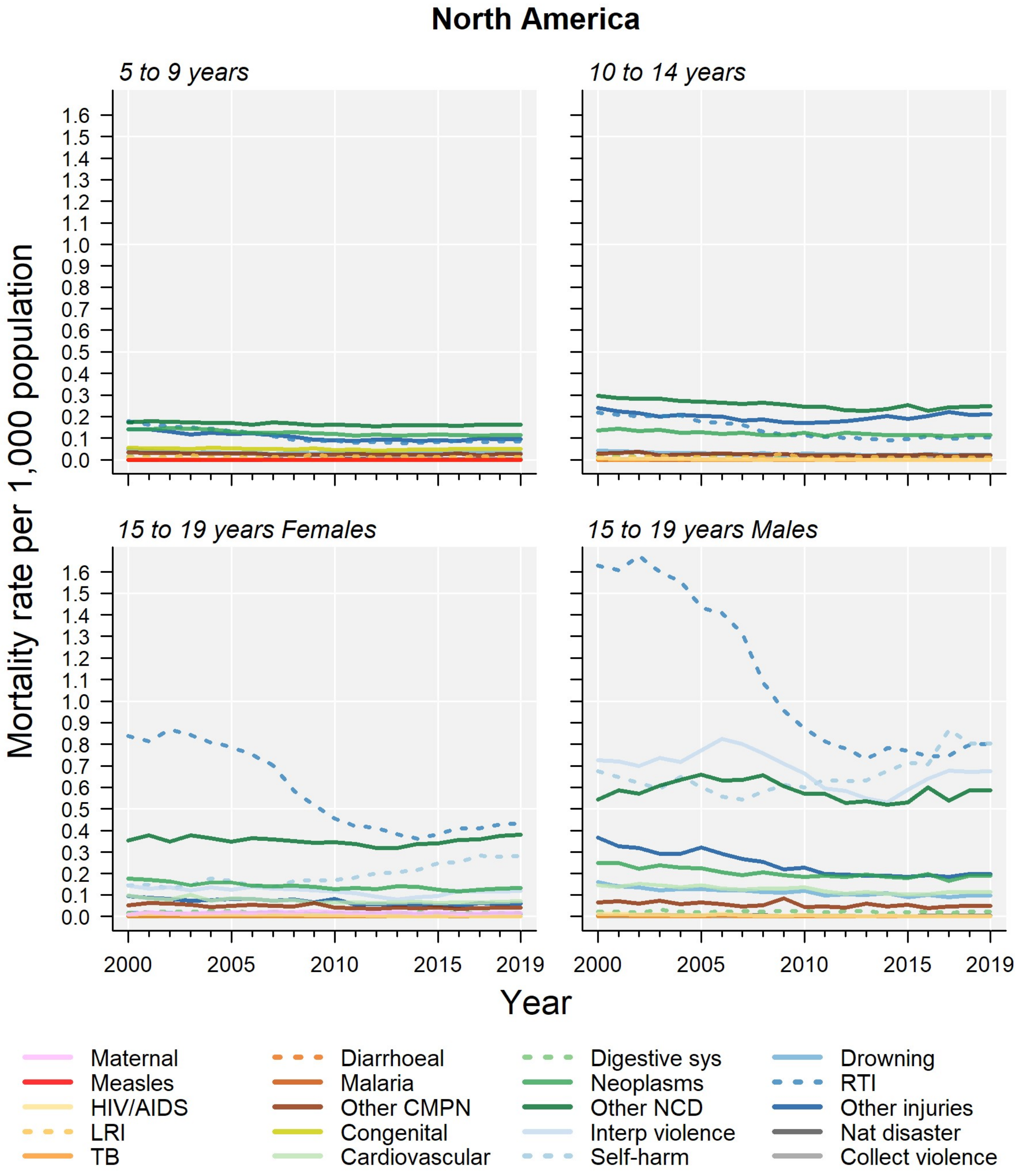
North America
5 to 9 years
10 to 14 years
15 to 19 years Females
15 to 19 years Males
Mortality rate per 1,000 population
1.6
1.5
1.4
1.3
1.2
1.1
1.0
0.9
0.8
0.7
0.6
0.5
0.4
0.3
0.2
0.1
0.0
2000
2005
2010
2015
2019
Year
Maternal
Measles
HIV/AIDS
LRI
TB
Diarrhoeal
Malaria
Other CMPN
Congenital
Cardiovascular
Digestive sys
Neoplasms
Other NCD
Interp violence
Self-harm
Drowning
RTI
Other injuries
Nat disaster
Collect violence

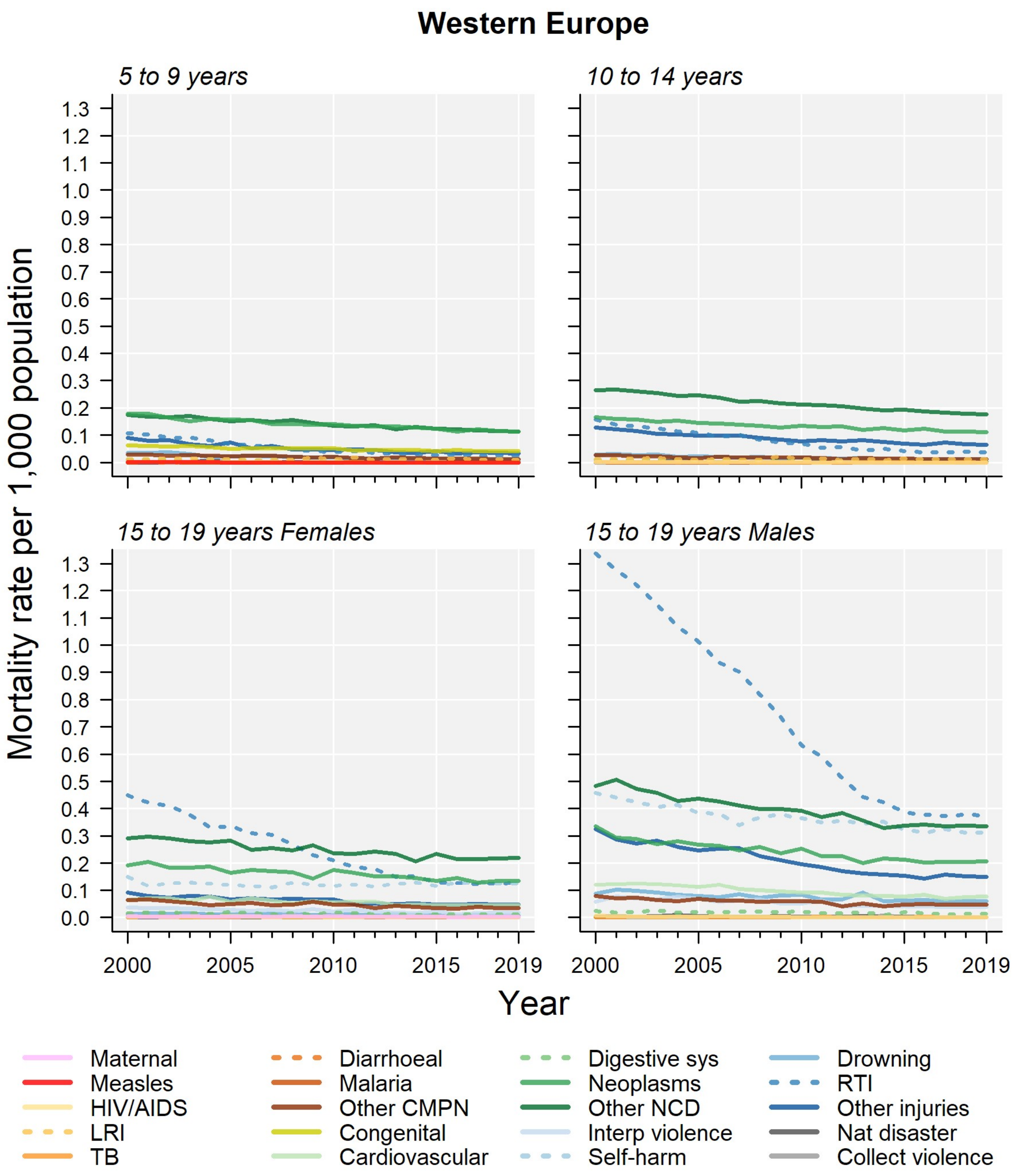
Western Europe
5 to 9 years
10 to 14 years
15 to 19 years Females
15 to 19 years Males
Mortality rate per 1,000 population
Year
Maternal
Measles
HIV/AIDS
LRI
TB
Diarrhoeal
Malaria
Other CMPN
Congenital
Cardiovascular
Digestive sys
Neoplasms
Other NCD
Interp violence
Self-harm
Drowning
RTI
Other injuries
Nat disaster
Collect violence

Webappendix 11. National cause-specific mortality estimates by age-sex group, 2000-2019

Webappendix 11.1 Data files with national cause-specific mortality estimates

The following CSV (comma separated value) files with mortality estimates are available from the GitHub repository https://github.com/panchoVG/Mort5to19.

Mortality estimates without uncertainty (point estimates)

- ‘PointEstimates5to9-National.csv’: National all-cause number of deaths, all-cause mortality rates, and cause-specific mortality fractions for 5-9 years, 2000-2019.
- ‘PointEstimates10to14-National.csv’: National all-cause number of deaths, all-cause mortality rates, and cause-specific mortality fractions for 10-14 years, 2000-2019.
- ‘PointEstimates15to19-National.csv’: National all-cause number of deaths, all-cause mortality rates, and cause-specific mortality fractions for 15-19 years, 2000-2019.
- ‘PointEstimates5to19-National.csv’: National all-cause number of deaths, all-cause mortality rates, and cause-specific mortality fractions for 5-19 years, 2000-2019.

Mortality estimates with 95% uncertainty intervals

- ‘AllCauseUncert-National.csv’: National all-cause number of deaths and all-cause mortality rates with 95% uncertainty intervals, 5-9, 10-14 and 15-19 years, 2000-2019.
- ‘Uncertainty5to9-National.csv’: National cause-specific number of deaths, mortality rates, and mortality fractions with 95% uncertainty intervals for 5-9 years, 2000-2019.
- ‘Uncertainty10to14-National.csv’: National cause-specific number of deaths, mortality rates, and mortality fractions with 95% uncertainty intervals for 10-14 years, 2000-2019.
- ‘Uncertainty15to19-National.csv’: National cause-specific number of deaths, mortality rates, and mortality fractions with 95% uncertainty intervals for 15-19 years, 2000-2019.

Webappendix 11.2 Countries with the highest number of deaths in 2019

Table S11.2. Top 10 countries with highest burden in 2019

#	ISO3	WHO name	5 to 19	5 to 9	10 to 14	15 to 19 females	15 to 19 males
1	IND	India	233,892 (199,891-270,701)	65,413 (54,728-76,120)	68,681 (43,872-98,054)	49,255 (39,237-59,623)	50,542 (40,262-61,180)
2	NGA	Nigeria	157,340 (120,584-197,615)	80,995 (65,229-99,534)	39,993 (11,042-73,632)	16,935 (11,045-25,681)	19,419 (12,664-29,446)
3	COD	Democratic Republic of the Congo	91,675 (67,070-120,212)	36,530 (26,147-48,208)	22,061 (5,149-40,831)	14,601 (8,102-22,917)	18,483 (10,255-29,009)
4	PAK	Pakistan	66,598 (41,809-111,674)	22,793 (15,134-30,080)	17,567 (0-61,117)	11,299 (6,056-18,007)	14,939 (8,008-23,810)
5	CHN	China	54,593 (42,895-73,731)	16,167 (10,492-22,256)	16,111 (8,140-31,604)	8,589 (5,718-12,145)	13,726 (9,138-19,409)
6	ETH	Ethiopia	51,279 (31,852-73,339)	17,497 (9,623-26,374)	13,204 (0-30,407)	8,020 (4,387-12,448)	12,558 (6,870-19,491)
7	IDN	Indonesia	45,934 (33,659-64,431)	14,772 (10,584-18,942)	9,017 (1,404-22,543)	8,273 (4,970-12,561)	13,872 (8,333-21,060)
8	BGD	Bangladesh	43,807 (32,428-61,188)	13,77 (10,811-17,825)	10,064 (1,596-24,079)	9,692 (6,678-13,725)	10,574 (7,286-14,974)
9	UGA	Uganda	32,001 (23,263-42,270)	11,937 (7,963-16,175)	6,399 (242-13,316)	5,195 (3,353-7,736)	8,470 (5,467-12,612)
10	TZA	United Republic of Tanzania	27,562 (19,425-48,491)	13,804 (8,500-17,571)	5,009 (0-25,427)	3,587 (2,191-5,506)	5,162 (3,152-7,922)

Note: Data are number of deaths (95% uncertainty intervals).

Webappendix 11.3 Cause-specific mortality rates by age-sex group in India, Nigeria and Democratic Republic of Congo, time trends 2000-2019

Time trends of the cause-specific mortality rates for the three countries with highest burden in 2019.

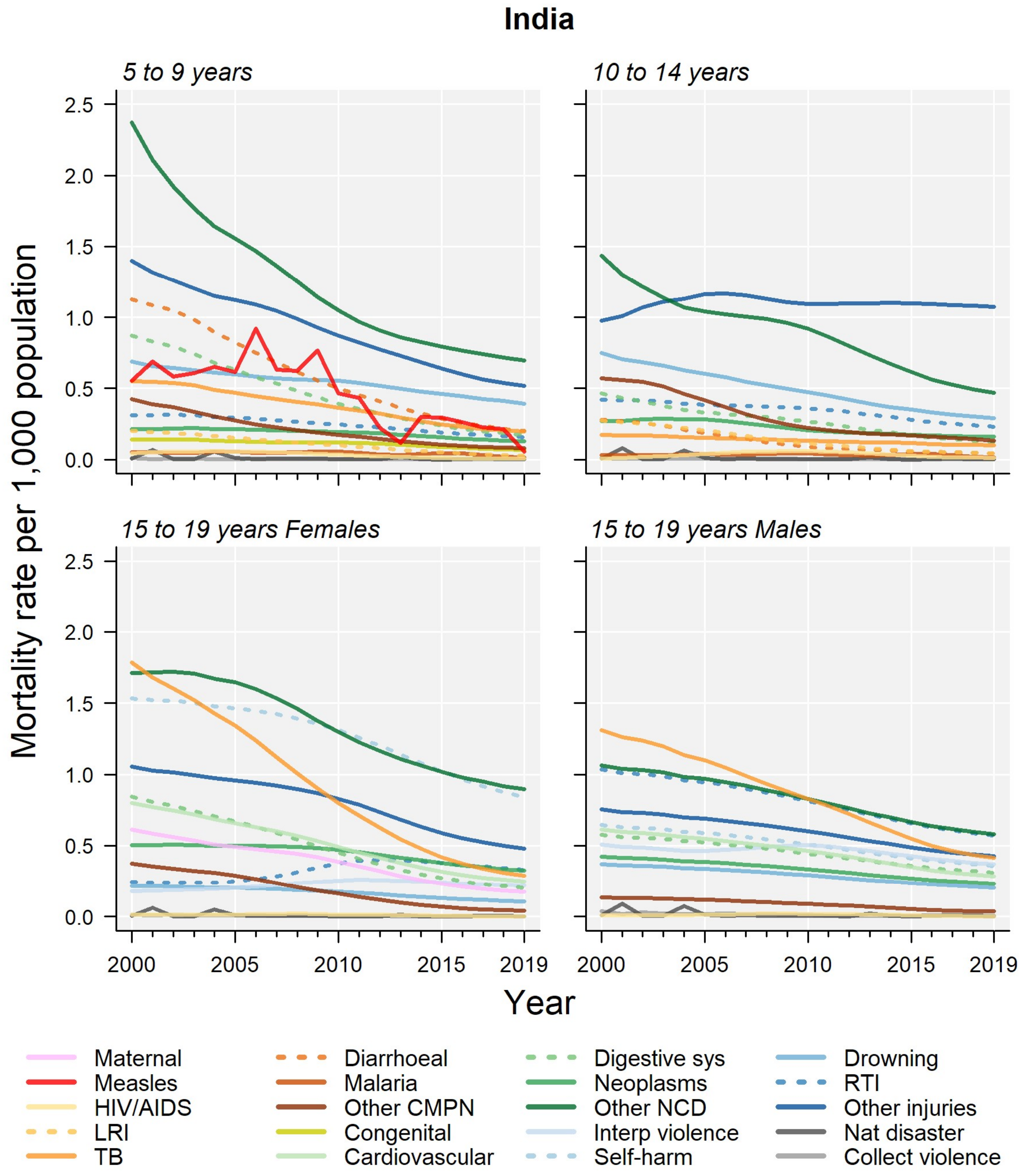

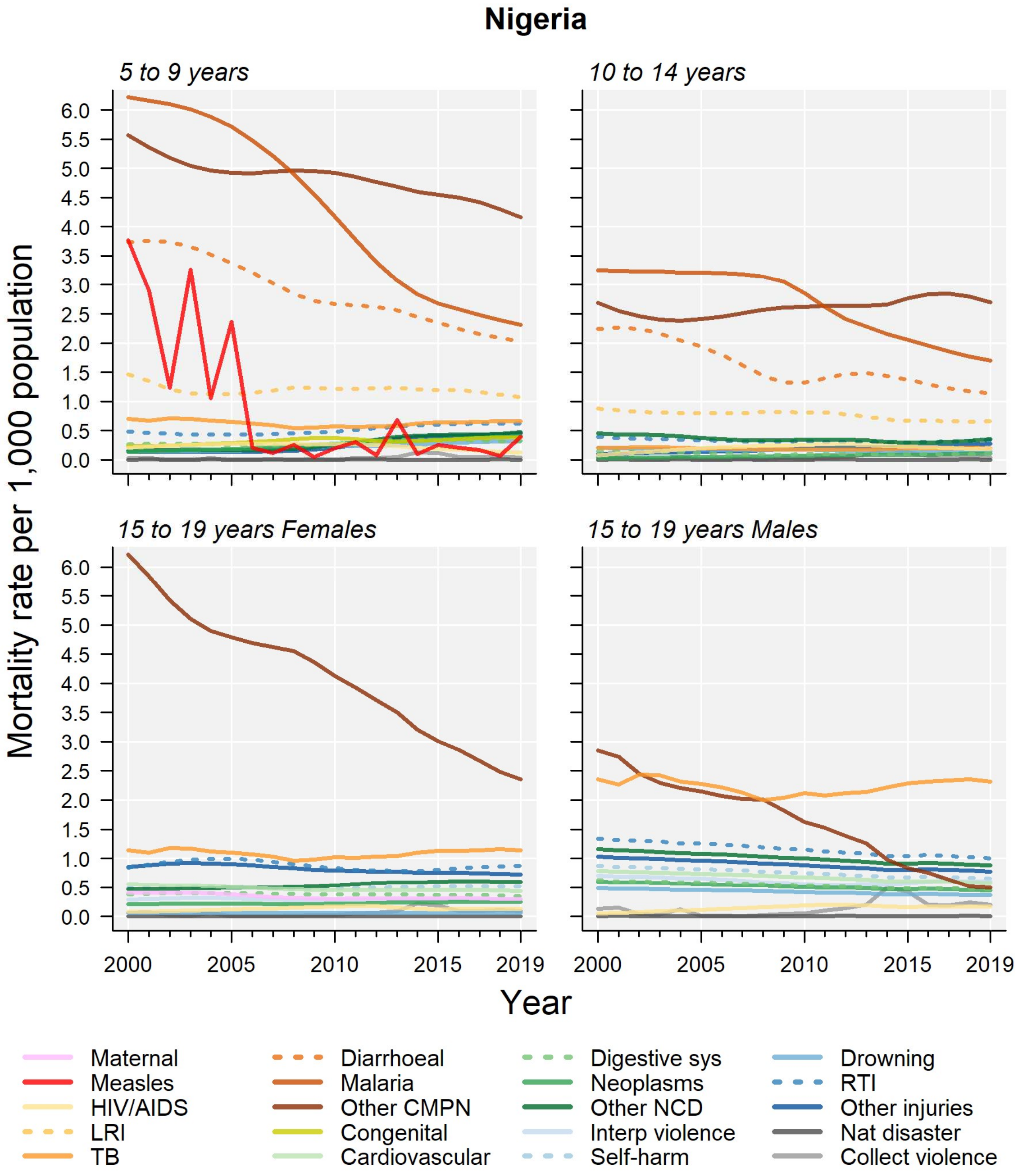
Nigeria
5 to 9 years
10 to 14 years
15 to 19 years Females
15 to 19 years Males
Mortality rate per 1,000 population
6.0
5.5
5.0
4.5
4.0
3.5
3.0
2.5
2.0
1.5
1.0
0.5
0.0
2000
2005
2010
2015
2019
Year
Maternal
Measles
HIV/AIDS
LRI
TB
Diarrhoeal
Malaria
Other CMPN
Congenital
Cardiovascular
Digestive sys
Neoplasms
Other NCD
Interp violence
Self-harm
Drowning
RTI
Other injuries
Nat disaster
Collect violence

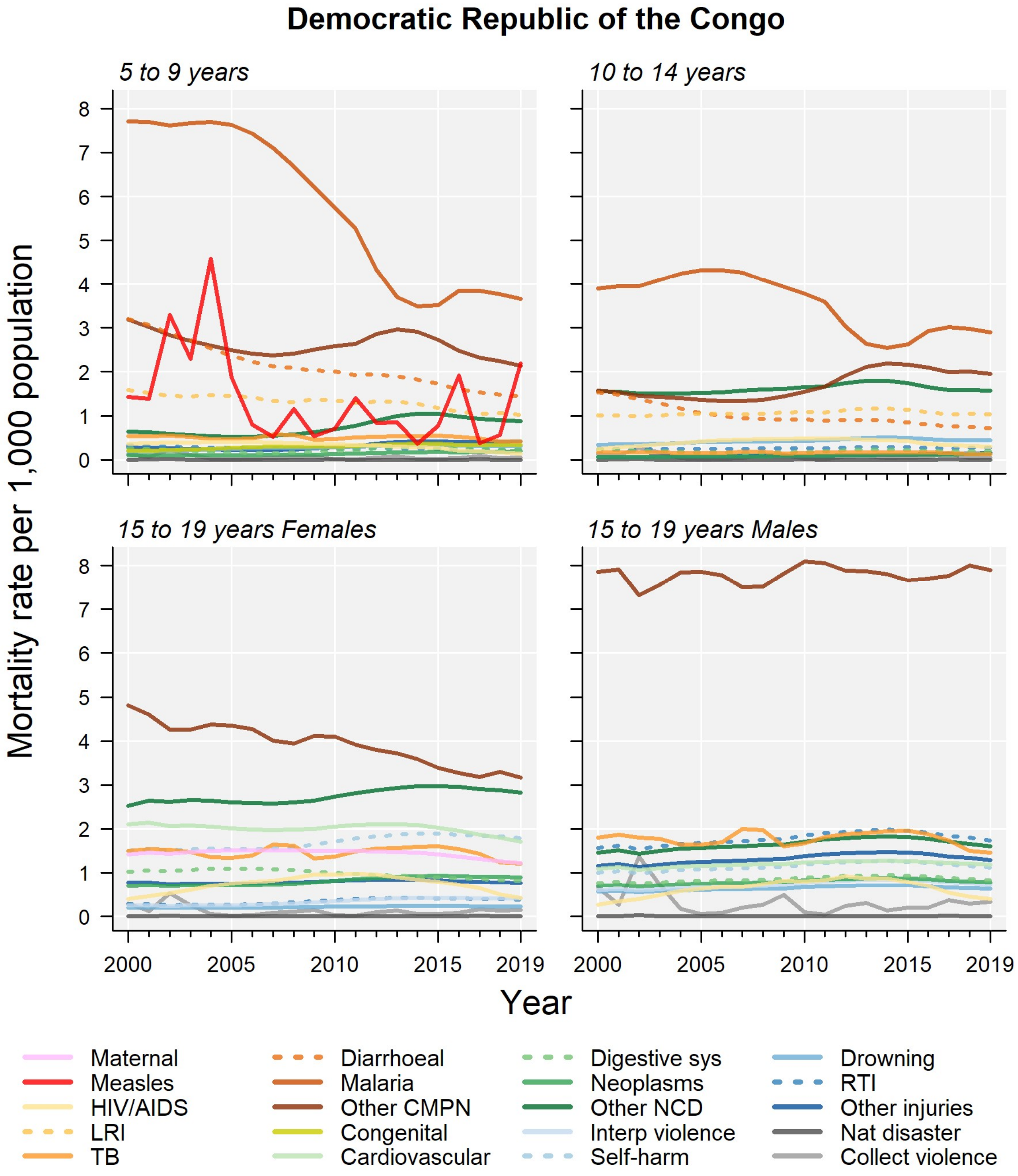
Democratic Republic of the Congo
5 to 9 years
10 to 14 years
15 to 19 years Females
15 to 19 years Males
Mortality rate per 1,000 population
Year
0
1
2
3
4
5
6
7
8
2000
2005
2010
2015
2019
Maternal
Measles
HIV/AIDS
LRI
TB
Diarrhoeal
Malaria
Other CMPN
Congenital
Cardiovascular
Digestive sys
Neoplasms
Other NCD
Interp violence
Self-harm
Drowning
RTI
Other injuries
Nat disaster
Collect violence

References

1. World Health Organization. Department of Data and Analytics (DNA), Division of Data, Analytics and Delivery for Impact (DNI). WHO methods and data sources for country-level causes of death 2000-2019. Geneva, 2020.
2. United Nations Inter-agency Group for Child Mortality Estimation (UN IGME). Levels and trends in child mortality. Report 2020, Estimates developed by the United Nations Inter-agency Group for Child Mortality Estimation. New York, NY: United Nations Children's Fund, 2020.
3. Liu L, Oza S, Hogan D, et al. Global, regional, and national causes of under-5 mortality in 2000-15: an updated systematic analysis with implications for the Sustainable Development Goals. *Lancet* 2016; **388**(10063): 3027-35.
4. WHO. Global Health Estimates 2016: Deaths by cause, age, sex, by country and by region, 2000-2016. Geneva: World Health Organization, 2018.
5. GBD 2017 Cause of Death Collaborators. Global, regional, and national age-sex-specific mortality for 282 causes of death in 195 countries and territories, 1980-2017: a systematic analysis for the Global Burden of Disease Study 2017. *Lancet* 2018; **392**(10159): 1736-88.
6. Stevens GA, Finucane MM, Paciorek CJ, et al. Trends in mild, moderate, and severe stunting and underweight, and progress towards MDG 1 in 141 developing countries: a systematic analysis of population representative data. *Lancet* 2012; **380**(9844): 824-34.
7. Buchard V, Randles C, Da Silva A, et al. The MERRA-2 aerosol reanalysis, 1980 onward. Part II: Evaluation and case studies. *Journal of Climate* 2017; **30**(17): 6851-72.
8. DistillerSR: A web-based systematic review product. https://www.evidencepartners.com/products/distillersr-systematic-review-software (accessed Apr 14, 2021).
9. Mulick AR, Oza S, Prieto-Merino D, Villavicencio F, Cousens S, Perin J. A Bayesian hierarchical model with integrated covariate selection and misclassification matrices to estimate neonatal and child causes of death. *medRxiv* 2021; published online Feb 2021. https://doi.org/10.1101/2021.02.10.21251488 (preprint).
10. Park T, Casella G. The Bayesian Lasso. *J Am Stat Assoc* 2008; **103**(482): 681-6.
11. Masquelier B, Hug L, Sharrow D, et al. Global, regional, and national mortality trends in youth aged 15–24 years between 1990 and 2019: a systematic analysis. *Lancet Glob Heath* 2021; **9**(4): e409-17.
12. United Nations Department of Economic and Social Affairs. World Population Prospects 2019. 2019. https://population.un.org/wpp/ (accessed Apr 24, 2021).
13. Gelman A, Carlin JB, Stern HS, Dunson DB, Vehtari A, Rubin DB. Bayesian data analysis 3rd edition. New York, NY: Chapman and Hall/CRC; 2013.
14. R Core Team. R: A language and environment for statistical computing. Vienna: R Foundation for Statistical Computing; 2021. URL: https://www.r-project.org/.
15. Plummer M. JAGS: A program for analysis of Bayesian graphical models using Gibbs sampling. 3rd International Workshop on Distributed Statistical Computing. 2003. https://www.r-project.org/conferences/DSC-2003/Proceedings/ (accessed Feb 27, 2019).
16. Su YS, Yajima M. R2jags: Using R to run 'JAGS'. R package version 0.7-1. 2020. https://cran.r-project.org/web/packages/R2jags/ (accessed Feb 23, 2021).
17. Simons E, Ferrari M, Fricks J, et al. Assessment of the 2010 global measles mortality reduction goal: results from a model of surveillance data. *Lancet* 2012; **379**(9832): 2173-8.
